# Wearable electronics for skin wound monitoring and healing

**DOI:** 10.20517/ss.2022.13

**Published:** 2022-06-30

**Authors:** Shubham Patel, Faheem Ershad, Min Zhao, Roslyn Rivkah Isseroff, Bin Duan, Yubin Zhou, Yong Wang, Cunjiang Yu

**Affiliations:** 1 Department of Engineering Science and Mechanics, The Pennsylvania State University, University Park, PA 16802, USA.; 2 Department of Biomedical Engineering, The Pennsylvania State University, University Park, PA 16802, USA.; 3 Department of Dermatology, University of California Davis, Sacramento, CA 95816, USA.; 4 Mary & Dick Holland Regenerative Medicine Program and Division of Cardiology, Department of Internal Medicine and Department of Surgery, University of Nebraska Medical Center, Omaha, NE 68198, USA.; 5 Center for Translational Cancer Research, Institute of Biosciences and Technology, Texas A&M University, Houston, TX 77030, USA.; 6 Department of Materials Science and Engineering, Materials Research Institute, Pennsylvania State University, University Park, PA 16802, USA.

**Keywords:** Wearable electronics, sensors, wound monitoring, wound healing

## Abstract

Wound healing is one of the most complex processes in the human body, supported by many cellular events that are tightly coordinated to repair the wound efficiently. Chronic wounds have potentially life-threatening consequences. Traditional wound dressings come in direct contact with wounds to help them heal and avoid further complications. However, traditional wound dressings have some limitations. These dressings do not provide real-time information on wound conditions, leading clinicians to miss the best time for adjusting treatment. Moreover, the current diagnosis of wounds is relatively subjective. Wearable electronics have become a unique platform to potentially monitor wound conditions in a continuous manner accurately and even to serve as accelerated healing vehicles. In this review, we briefly discuss the wound status with some objective parameters/biomarkers influencing wound healing, followed by the presentation of various novel wearable devices used for monitoring wounds and accelerating wound healing. We further summarize the associated device working principles. This review concludes by highlighting some major challenges in wearable devices toward wound healing that need to be addressed by the research community.

## INTRODUCTION

Skin is the largest organ in the human body, which acts as an important barrier to protect internal organs from the external environment. However, various factors, such as physical damage, whether intentional (surgery) or unintentional (abrasions, burns, etc.), and diseases can threaten the integrity of the skin tissue. A break or defect of skin tissue is called a wound, and a wound that does not heal in the normal time frame is defined as a chronic wound. The lack of therapies that hasten the healing of chronic wounds can result in further wound complications, including infection and even amputation^[1,2]^. Therefore, timely and effective management of chronic wounds is crucial to accelerate wound healing and relieve the patient’s pain. Current approaches to wound care rely on traditional wound dressings that absorb excessive wound exudate, retain sufficient moisture, and provide a protecting barrier against the external environment^[3,4]^. Some of these dressings involve the embedding of anti-inflammatory drugs, antibiotics, antibacterial compounds, or angiogenic factors released into the wound^[5,6]^. These traditional methods of wound care have been very effective but have some limitations. One of these limitations is that these methods fail to indicate the status of the wounds’ bed and their healing rate due to a lack of real-time monitoring and can cause secondary injuries while being replaced. Typically, clinicians monitor and assess wounds subjectively by using the following or a combination of the following processes, including visual inspection, wound sampling, or bacterial culture. These processes can be invasive, time-consuming, and only provide data about the wound microbiome from the initial sampling. Furthermore, these processes may lead to misdiagnosis since, at the time of treatment, the wound microbiome may have changed as compared to the time of initial sampling.

To solve this problem, there is a need to detect the wound stage and provide a reliable real-time assessment of its status for timely treatment. A promising solution is to use highly conformal, flexible, biocompatible devices that are enabled for localized and remote real-time monitoring in the clinic or patient’s home for long-term duration. These devices not only provide objective diagnostic information about wound biomarkers with high sensitivity and stability, but also combat infection. These interventions may improve healing in chronic wounds by intervening in the wound healing process. Such intelligent devices, which can sense, respond, report, or have combinational functions, can address many problems associated with wound chronicity. These also allow for better wound management, improving clinical outcomes by early detection of infections.

**Figure 1.**
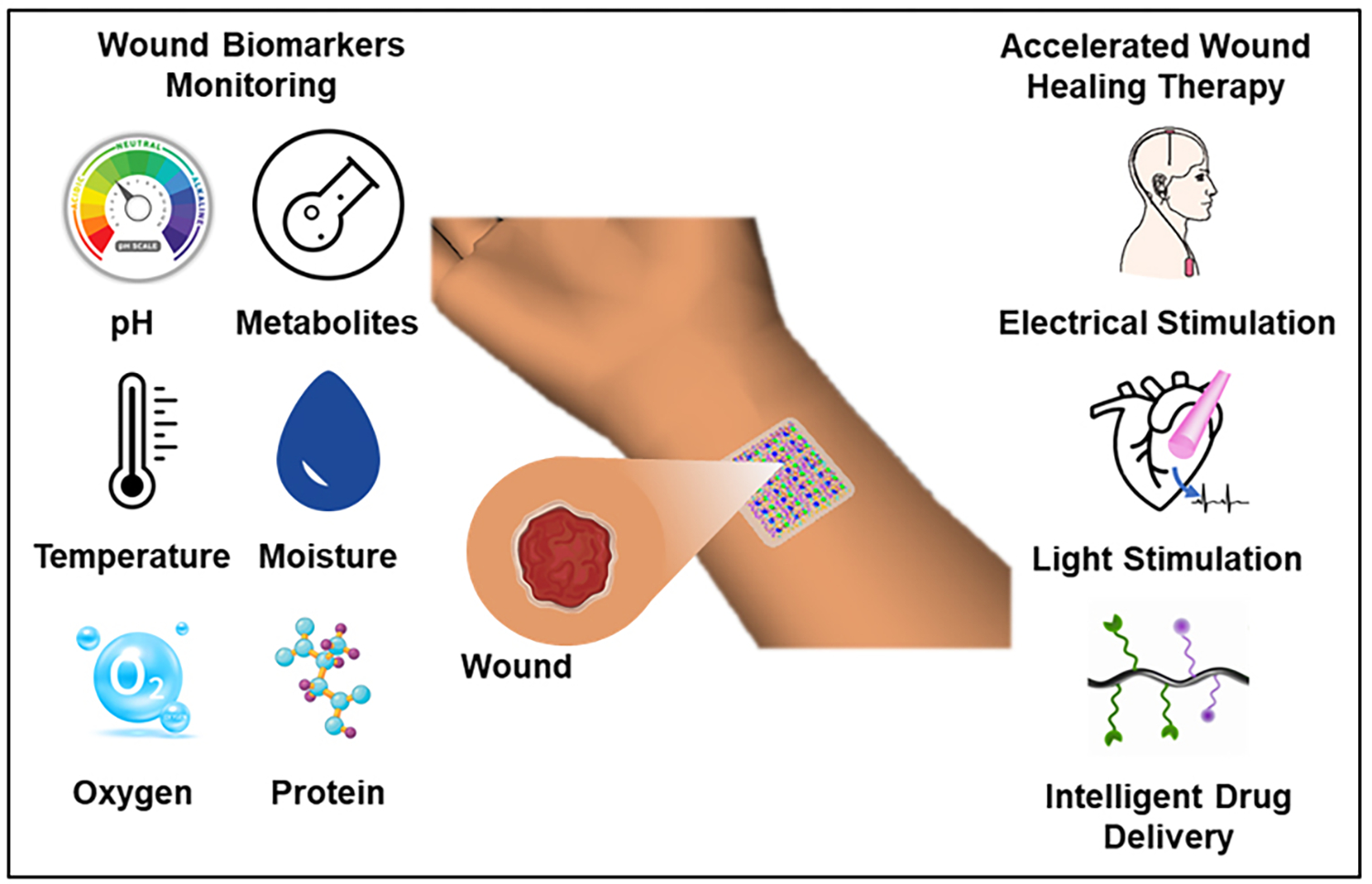
Schematic illustration of wearable devices for wound monitoring and healing.

This review provides a comprehensive overview of recent progress in wearable electronics for wound monitoring and wound healing [Figure 1]. The first section of this review address the wound healing mechanisms, along with the important physiological parameters/biomarkers influencing wound healing. The second section discuss wearable electronic devices/patches recently developed and researched for wound monitoring, considering various physiological parameters/biomarkers. In the next section, wearable devices for wound treatment are discussed, followed by a discussion on challenges and opportunities for wound care wearable electronics in the final section.

## WOUND HEALING MECHANISM AND BIOMARKERS

**Figure 2.**
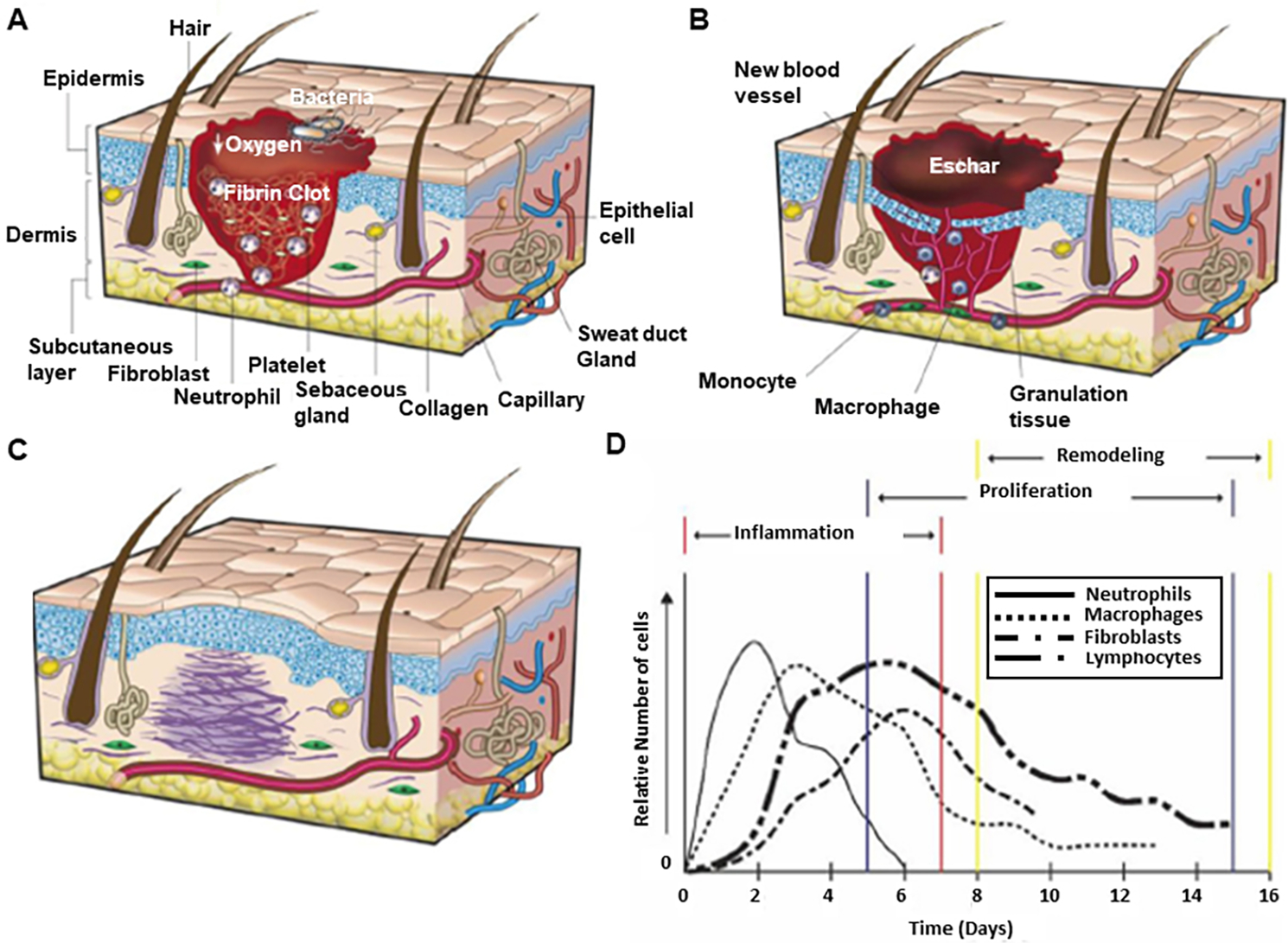
The wound healing mechanism. The three stages of wound healing: (A) hemostasis and inflammation (B) proliferation (C) remodeling. Reproduced with permission^[9]^. Copyright 2008, Nature. (D) The timeline of cell migration for immune cells during wound healing. Reproduced with permission^[10]^. Copyright 1997, W. B. Saunders Company. Published by Elsevier.

The wound healing of human skin is a complex process usually divided into three stages^[2,7,8]^: (1) the hemostasis and inflammation stage [Figure 2A]; (2) the proliferation stage [Figure 2B]; and (3) the remodeling stage [Figure 2C]^[9]^, overlapping in time and space [Figure 2D]^[10]^.

### (1) Hemostasis and Inflammation stage:

This stage includes the immediate initiation of hemostasis after tissue damage followed by constriction of blood vessels near the wound site, aggregation of blood platelets to form a platelet clot followed by the formation of fibrin matrix cleaved from fibrinogen during coagulation of blood^[2,8,11]^. The platelet plug and fibrin matrix formation leads to the release of cytokines and growth factors that cause an inflammatory reaction essential to tackle pathogens, bacteria, or toxins and initiate the repair process. Neutrophils initiate the inflammatory response immediately after arriving at the wound site. Then monocytes migrate into the wound, differentiating into macrophages. Macrophages, which differentiate along a spectrum of functions^[12]^ have at various phases of healing, play the roles of phagocytosis, supporting angiogenesis, cell recruitment and activation, removal of wound debris, and synthesis of wound matrix^[2,13]^. Some populations of wound macrophages synthesize nitric oxide, which has antimicrobial properties, induces angiogenesis, and increases collagen deposition, ultimately improving scar mechanical strength.

### (2) Proliferation stage:

Keratinocytes, fibroblasts, lymphocytes, myofibroblasts, and endothelial cells are involved in the proliferation stage. Keratinocytes aggregate at the edge of the wound and migrate to cover the wound bed to restore the skin’s barrier function^[14,15]^. Fibroblasts become activated in the surrounding tissue, resulting in their proliferation and migration to the wound site. Then, they secrete collagen, fibrinogen, and extracellular matrix (ECM) to replace the fibrin matrix^[14]^. This leads to the formation of granulation tissue, which is essential for tissue repair and angiogenesis. New blood vessels are then formed through angiogenesis and create new capillaries from existing blood vessels. This process is critical as it provides the wound site with nutrients and oxygen that are crucial for cell proliferation. Epithelial cells proliferate in the wound to reestablish a barrier to the external environment to prevent fluid loss and infection^[10,15]^.

### (3) Remodeling stage:

In this stage, the processes initiated by the immune/inflammatory response slow down and are then stopped. The macrophages and neutrophils undergo apoptosis, and collagen and protein ECM formation continue in the wound bed^[10]^.

The approximate sequence for normal healing of an acute wound and the attendant migration of immune cells during wound healing is shown in Figure 2D.

The acute wound can become chronic if the inflammatory stage is prolonged, delaying the progress of wound healing. In such a case, proinflammatory cytokines such as interleukin-6 (IL-6), interleukin-1 (IL-1), etc., continue to be secreted by neutrophils and macrophages, which causes a delay in healing progress^[16,17]^. IL-6 elicits the release of monocyte chemoattractant protein-1 and monocyte chemoattractant protein-2, thus recruiting monocytes for maintaining the inflammatory response and modifying the immune system, respectively. Also, proinflammatory cytokines lead to the elevated release of metalloproteinases from neutrophils that degrade the ECM, thus impeding cell migration to the wound site^[2,17]^. Due to this, neutrophils and macrophages remain at the wound site, continuously secreting proinflammatory cytokines, thus prolonging the inflammatory stage. If the chronic wound passes the inflammatory stage and enters the proliferation stage, hypoxia at the wound site may slow down the healing progress. Chronic wounds are considered ideal for developing biofilms as necrotic tissues allow suitable bacterial attachment. Once attached to the wound site, bacteria multiply, develop microcolonies, and produce a substance called extracellular polymeric substances, similar to ECM, to create a biofilm^[1,2,18]^. Once the biofilm is developed, it leads to persistent infections, which are harder to eradicate, and these bacteria have a high tolerance to antibiotic treatment. Biofilms also consume a lot of nutrients^[19–21]^, further impairing the immune response to combating infections and regenerating tissues, thus prolonging the wound healing process.

There are many biomarkers to indicate more delicate progress of wound healing. These also include a collection of inflammatory biomolecules. Some important biomarkers and their influence on wound healing are discussed below.

#### pH:

The pH of healthy human skin ranges from 4–6, slightly acidic to prevent bacterial growth^[22,23]^. The skin’s acidic environment gets disturbed when a wound develops on the skin. pH plays a crucial role in wound healing; thus, pH measurements of wounds can help identify chronic wounds early in the wound healing process. The pH of the wound can be detected from wound fluids/exudate, and the different pH values can be an indicator of the various stages of wound healing^[24]^. Acute wounds tend to be slightly acidic, 5–6 pH range like healthy skin^[24–26]^. Chronic wounds, however, tend to be in a basic environment, with pH ranging from 7–9, due to the proliferation of bacteria^[24–26]^. The degree of bacterial colonization or infection in the wound may alter wound pH.

#### Temperature:

At the wound site, temperature measurement can provide information regarding local blood flow^[27]^ and wound infection and its healing progress. Increased temperature is associated with the inflammation stage, but a prolonged increase in temperature of at least 1.11 °C can also indicate bacterial infection and metabolic activity changes^[23,28]^. A decrease in temperature at the wound site can indicate the presence of partial ischemia, threatening the wound healing progress. Also, a correlation has been found between increased temperature and increased angiogenesis and fibrosis^[29,30]^.

#### Glucose:

Glucose metabolism generates adenosine triphosphate, driving many thermodynamically unfavorable reactions in the body by glycolysis. Average glucose concentrations present in the blood range from 3.9 to 7.8 mM^[31]^, where higher glucose levels may indicate a deficiency in insulin. Abnormal insulin levels may indicate the presence of diseases like diabetes mellitus, thus resulting in increased glucose sensitivity. For patients with this disease, skin injury may result in a non-healing wound with increased chances of amputation^[32]^. Higher glucose levels can impede wound healing by slowing growth factor production, angiogenesis, macrophage function, collagen deposition, and fibroblast aggregation at the wound; scar strength is also lowered^[33]^.

#### Uric Acid:

It has been found that elevated uric acid levels correlate to wound severity and oxidative stress in venous leg ulcers^[34]^. Elevated uric acid levels prolong the inflammation stage of wound healing, thus inhibiting the wound healing progress. Also, the production of uric acid has been associated with a burst of highly reactive superoxide radicals and subsequent generation of another redox regulating reagent, H_2_O_2_^[34]^. Excessive release of these oxidants can alter the structure of lipids, DNA, and proteins, thus, disrupting the normal wound healing process.

#### Oxygen:

It is required for collagen deposition, epithelialization, fibroplasia, angiogenesis, infection resistance, etc.^[35]^. Without oxygen, tissue hypoxia occurs, which causes prolonging of the wound healing process^[36]^. The partial pressure of oxygen in non-healing/chronic wound exudate is 5–20 mmHg during prolonged inflammation, whereas in healthy tissues, it is around 30–50 mmHg^[37]^.

#### Moisture:

A dry or very wet wound slows down the wound healing process. Dry wounds are not suitable for cell growth. Cells need an appropriate moist environment to grow, divide, and migrate to enhance wound healing^[38]^. Hence, dry wounds have a slower progression to the remodeling stage of wound healing. However, excessive moisture/exudate also impairs wound healing, as some bacterial species grow better in a moist environment, so excessive moisture may increase the risk of bacterial infection^[30,39]^.

#### Proteins:

Many proteins are secreted by cells that participate in the wound healing process. Proinflammatory cytokines like tumor necrosis factor (TNF), IL–6, etc., are proteins of relatively low molecular weight that are among the first to be secreted by cells like macrophages, neutrophils, etc. on the onset of wound that plays a critical role in the inflammation stage of wound healing by exerting cascades of inflammatory reactions and prevent wound infection^[40]^. However, an excess of proinflammatory cytokines may prolong the inflammation stage of wound healing, indicating that the wound is chronic. Elevated metalloproteinases due to proinflammatory cytokines have been shown to degrade the ECM, diminishing cell migration at the wound site^[17]^. Hence, real-time monitoring of proinflammatory cytokines is important to monitor the stage of the wound, whether the wound is acute or chronic. Another protein that can be commonly used as a biomarker for wound healing is C-reactive protein, which is a member of the acute phase family of proteins, released mainly from liver hepatocytes whose level rise significantly in response to proinflammatory cytokines (IL-1, IL-6, etc.) because of wound infection, tissue injury, etc.^[41,42]^.

## WEARABLE ELECTRONICS FOR SKIN WOUND MONITORING

### pH:

There are several methods to determine the pH value of wound beds ranging from optical to electrochemical methods. In optical methods, pH sensors typically use chemical species that modify their optical properties as a function of pH. Electrochemical pH sensors are mainly potentiometric and voltammetric. However, the potentiometric measurement is the preferred choice for pH wound monitoring due to its high reliability and wide sensing range. Potentiometric sensors measure the potential generated across two electrodes, i.e., the working electrode and the reference electrode.

**Figure 3.**
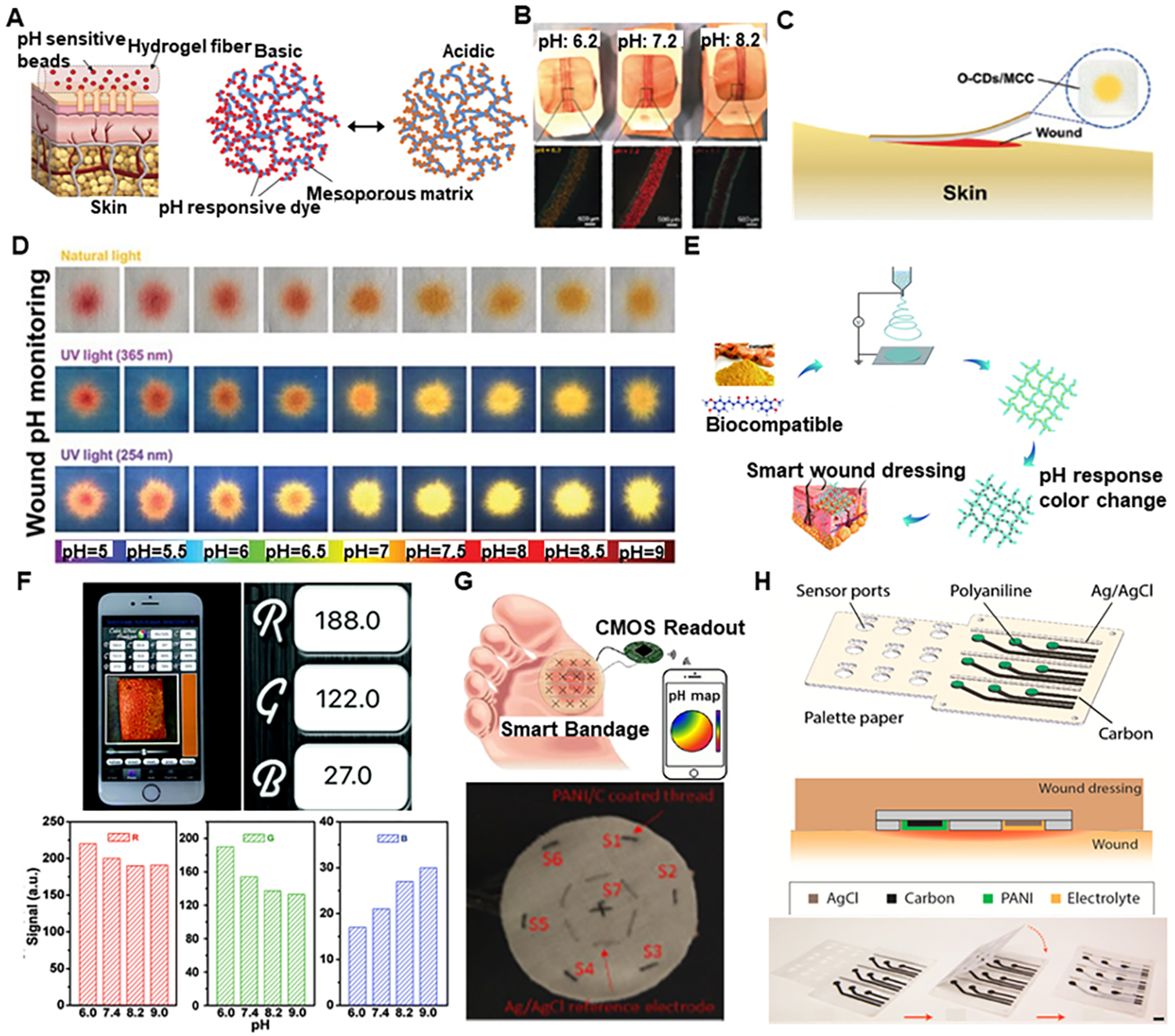
Wearable pH sensing devices for wound healing. (A) (i) Schematic illustration of pH-sensing hydrogel microfibers designed for long-term epidermal monitoring; (ii) Action Mechanism of the mesoporous polyester particles containing pH-responsive dye with electrostatic interaction to the solid matrix of mesoporous particles. (B) Fabricated wound dressings laced on pieces of pig skin sprayed with solutions of different pH. Reproduced with permission^[45]^. Copyright 2016, WILEY-VCH Verlag GmbH & Co. KGaA, Weinheim. (C) Schematic and conceptual view of O-CDs/MCC in practical application. (D) Color appearance under natural light and fluorescence images under UV light (excited at 365 and 254 nm) of the O-CDs/MCC treated by buffer solution at different pH values. Reproduced with permission^[48]^. Copyright 2019, WILEY-VCH Verlag GmbH & Co. KGaA, Weinheim. (E) Schematic illustration of the fabrication process of curcumin-loaded polymer fibers and pH-responsive color changing. (F) RGB values determined using a free app on phone, and RGB values under different values of pH. Reproduced with permission^[49]^. Copyright 2019, Royal Society of Chemistry. (G) Conceptual and optical image of pH sensing smart bandage^[50]^. Copyright 2017, IEEE. (H) Schematic of 3 × 3 pH sensor array on paper with self encapsulation and optical image of the encapsulation process. Reproduced with permission^[51]^. Copyright 2016, published by Elsevier.

Optical pH sensors are mainly based on pH-sensitive dyes with specific pKa, high absorbance, and high emission spectra in the visible range. These pH-sensitive dyes show color changes depending on the pH and can be used to monitor pH variation. However, there are some challenges regarding adhesion between the dyes and dressing substrate^[43,44]^. Keeping this in mind, an attempt to make alginate-based hydrogel fibers incorporated with mesoporous particles loaded with pH-sensitive brilliant yellow dye [Figure 3A] were fabricated using a coaxial flow microfluidic spinning system^[45]^. The pH dyes can stably combine with the mesoporous matrix using electrostatic interaction, preventing dye leakage from fiber to wound. The pH response of the beads attached to the fibers is schematically shown in Figure 3A. These pH-sensitive hydrogel fibers were then assembled over a clear medical tape to create a wound dressing for long-term monitoring in the pH range of 5.5–9.0. The response of this hydrogel assembled over medical tape for exudates of various pH is shown in Figure 3B. Similarly, alginate-based hydrogel fibers loaded with mesoporous resin beads doped with pH-sensitive brilliant yellow were fabricated using a modified 3D printer with a micro-extruder mounted with two coaxial needles^[46]^ having a pH sensing range of 4.0–9.0. In another attempt using alginate fibers, smart pH-responsive, flexible sensors were prepared by modifying commercial calcium alginate fibers to enhance dying properties and then dyed with alizarin and anthocyanin dye, respectively^[47]^. They showed color change response in 2.0–11.0 pH range. Orange-emissive carbon quantum dots (O-CDs) were synthesized and then assembled with a medical cotton cloth (MCC) [Figure 3C]^[48]^, which changed color from orange to yellow as pH changes from 5.0 to 9.0 under natural light, as shown in Figure 3D. The visual fluorescence increased under excitation with 365 nm and 254 nm UV lamps as the pH changed from 5.0 to 9.0. The O-CDs were prepared through microwave-assisted heating of urea and 1,2,4-triaminobenzene solution at 200 °C. The surface of nanosized O-CDs has many different functional groups like hydroxyl, amino, and carboxyl, thus forming a strong hydrogen bond to MCC, preventing O-CDs from getting off. Another way of fabricating pH-responsive fibers is using the electrospinning technique, which improves wound monitoring sensitivity. Curcumin-loaded polycaprolactone fibrous mat was prepared by electrospinning for achieving in-situ pH monitoring [Figure 3E] with a pH range from 6.0 to 9.04^[49]^. The composite mat went through color changes during pH monitoring when exposed to a pH from 6.0 to 9.0. The RGB values can be extracted from the picture taken by the smartphone and then used to relate to pH values, with some results shown in Figure 3F.

For more precise detection, electrochemical sensors with innate selectivity and sensitivity towards target analytes are applied to transduce pH into potential, current, or impedance. A wireless pH sensing bandage with numerous pH sensing threads [Figure 3G] was designed for chronic wound monitoring^[50]^. The pH sensing threads were prepared by first immersing the cotton thread substrate in isopropanol, then through carbon resistive ink to make the thread conductive, and then finally through Polyaniline (PANI) ink to make them pH-sensitive, which are widely used for their ability of reversible protonation and deprotonation. Similarly, a reference Ag/AgCl reference thread was prepared by coating the thread in Ag/AgCl ink. Then, all functionalized threads were sewed into a commercial bandage for pH detection in a chronic wound. This system exhibited a high pH sensitivity of 54 mV/pH for pH sensing ranging from 2.0 to 10.0. Another similar attempt to prepare a potentiometric pH sensor was made by fabricating a flexible array of pH sensors on a palette paper, with each sensor consisting of two screen-printed electrodes, an Ag/AgCl reference electrode, and a carbon electrode coated with PANI membrane as sensing electrode [Figure 3H]^[51]^. Laser machining was used to create a self-passivation layer with access holes bonded over the sensing and reference electrodes. These sensors had a pH sensing range of 4.0–10.0 with a 50 mV/pH sensitivity. The designed sensors also proved their biocompatibility by showing good cell viability for human keratinocytes cells. In addition to the materials used above, some other pH sensors based on different sensing materials like graphite^[52]^, poly-L-tryptopan^[53]^, CuO nanorods^[54]^, etc., have been considered to be used in monitoring wound pH.

### Temperature:

**Figure 4.**
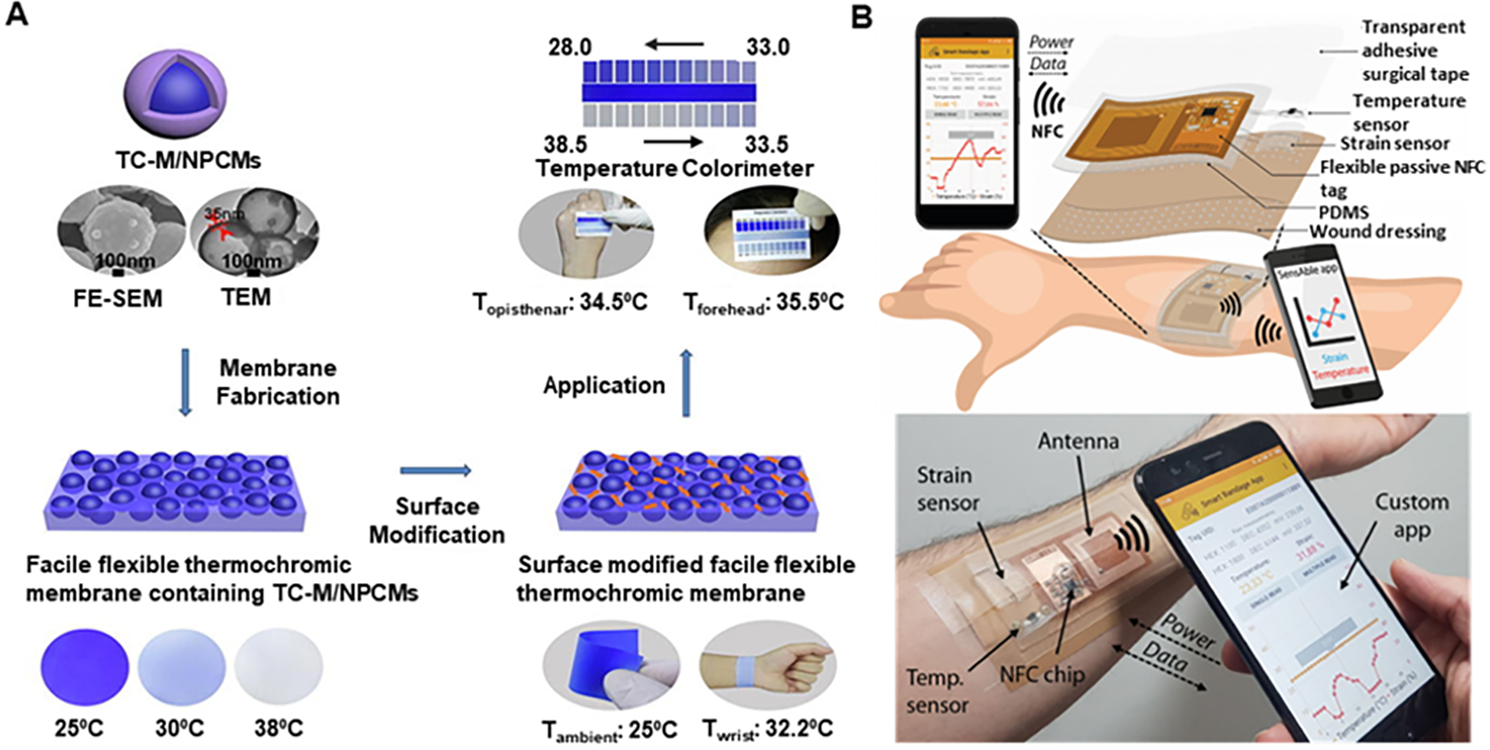
Wearable temperature sensing devices for wound healing. (A) Facile flexible reversible thermochromic membranes based on micro/nano-encapsulated phase change materials for the wearable temperature sensor. Reproduced with permission^[57]^. Copyright 2019, Elsevier. (B) Conceptual and optical image of NFC-based smart bandage attached to the arm for wireless temperature monitoring using a custom smartphone app. Reproduced under the terms and conditions of the CC BY 4.0^[59]^. Copyright 2020, The Author(s), published by IEEE.

The temperature of the wound environment can be determined using different sensors like infrared sensors and resistance temperature sensors. Infrared sensors-based portable temperature sensing devices are more often used to provide thermal imaging for clinical tissue structure monitoring for wound healing. Forward-looking infrared camera integrated with smartphones is widely used to produce thermal images with a 2-D resolution to improve diagnosis efficiency^[55]^. However, in the case of wound healing, there is a major limitation to this technology, which is the need to remove the wound dressing during the wound assessment by an infrared camera, thus increasing the probability of causing secondary injury to the patient. One way of avoiding such limitations is to use a visualization technique for in-situ real-time monitoring of wounds by conformal contact with the human skin. This can be achieved by using thermochromic materials, which change color with a change in temperature^[56]^. Using these thermochromic materials, a facile, flexible reversible polyvinyl alcohol (PVA)/water-soluble polyurethane (WPU) composite membrane containing thermochromic micro/nano encapsulated phase change materials (TC-M/NPCMs) was developed [Figure 4A]^[57,58]^. The colorimetric responses to different temperatures are shown in Figure 4A, indicating good sensitivity of the flexible composite membranes. These thermochromic membranes exhibit a dual-functional feature of effective temperature adjustment and reversible thermal indication. Hence, this thermochromic membrane has great potential for applications in wound monitoring.

Considering a resistive temperature sensor for wound healing, a smart bandage with wireless temperature sensor and battery-less NFC tag [Figure 4B]^[59]^ was fabricated with the temperature sensor consisting of a PEDOT:PSS deposition over a 2 mm gap between two Ag ink electrodes over a commercial PVC substrate^[56]^. The PEDOT:PSS-based temperature sensor showed a ~70% decrease in resistance for a temperature change from 25 °C to 90 °C with a sensitivity of ~1.2%/°C. Also, other materials like graphene^[60]^, MXene^[61]^, etc., have been used for developing resisitive temperature sensors for monitoring wound temperature. Another approach to temperature sensing is using capacitance. An LC (inductance-capacitance) oscillator with polyethylene glycol as the sensing material was developed to measure regional body temperature through a change in capacitance to measure a temperature range of 34 °C-42 °C^[62]^.

### Glucose:

The basic concept of a glucose sensor (biosensor) is based on the fact that the immobilized glucose oxidase (GO_x_) catalyzes the oxidation of glucose by molecular oxygen producing gluconic acid and hydrogen peroxide. This is the common reaction for nearly all glucose sensors based on GO_x_ enzyme-based sensing. Most of the studies done for glucose sensing for wound healing are based on fluorescent glucose sensing. A fluorescence glucose sensor molecule consists of a receptor for glucose, a donor fluorophore, and an acceptor of fluorescent energy or electrons. When glucose gets bonded to the receptor, the sensor molecule undergoes a structural change that brings the fluorescence donor and acceptor further apart, decreasing the electron transfer to the donor. Binding glucose to this type of receptor-fluorophore molecule can decrease fluorescence resonance energy transfer, leading to a decrease in electron sharing and increasing fluorescence. Also, in the absence of glucose, the fluorophore donor and acceptor develop increased electronic interactions resulting in increased transfer of electrons to the donor fluorophore and less fluorescence due to the increased amount of fluorescence resonance energy transfer between these groups. The glucose concentration can be detected by measuring any change from baseline in fluorescence of the donor fluorophore associated with the glucose receptor molecule^[63]^. Using this mechanism, wearable glucose sensors for wound monitoring have been developed.

**Figure 5.**
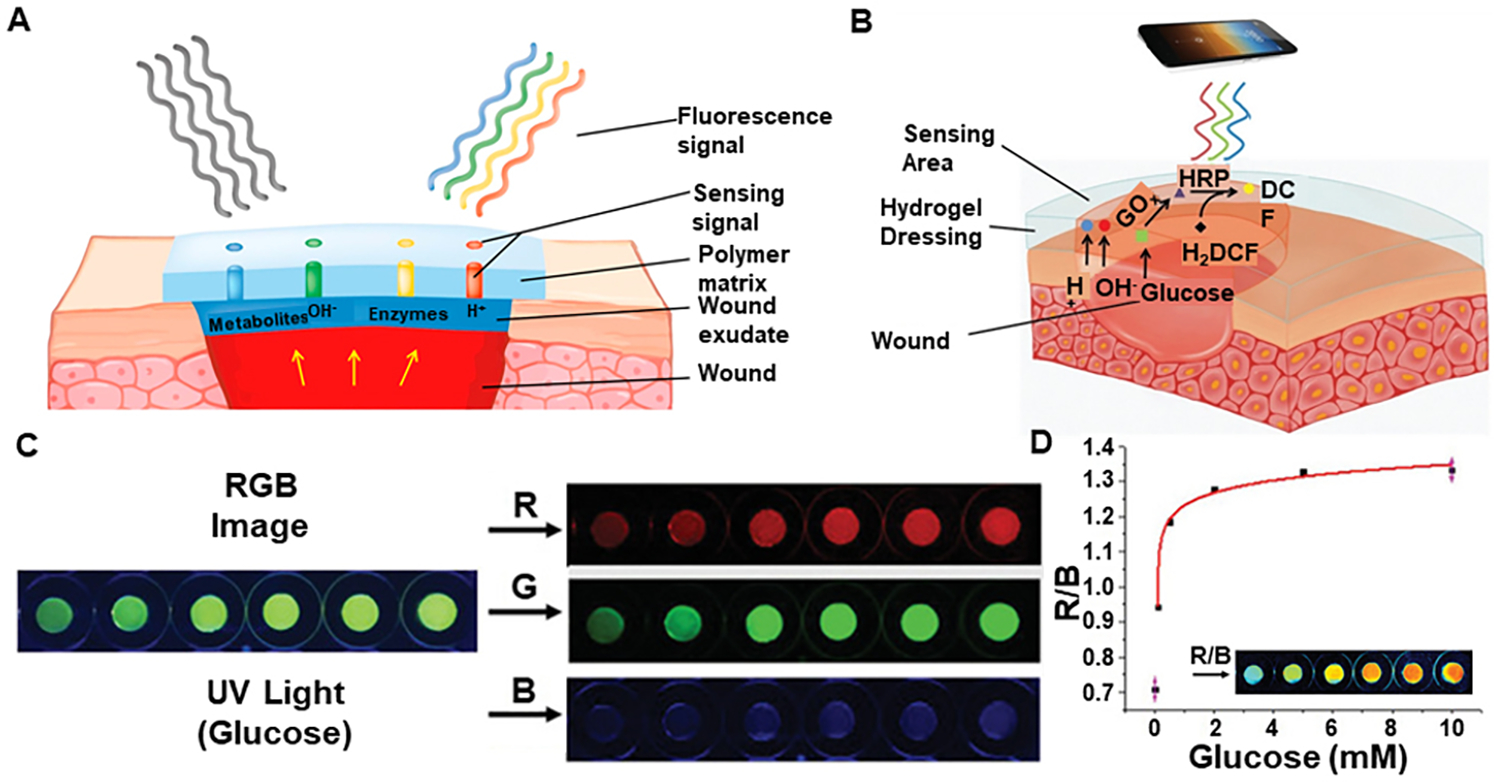
Wearable glucose sensing devices for wound healing. (A) Schematic diagram of polysaccharide matrix-based hydrogel with immobilized pH indicator dye and glucose-sensing enzymatic system. Reproduced with permission^[64]^. Copyright 2016, Elsevier. (B) Schematic illustration of PCB hydrogel dressing for pH and glucose detection (C) RGB images of a PCB-PE-E hydrogel sensor with different glucose concentrations. (D) Representative fitted curve for glucose concentration with inset showing R/B pictures displayed in pseudo colors representing glucose sensing. Reproduced with permission^[65]^. Copyright 2019, WILEY-VCH Verlag GmbH & Co. KGaA, Weinheim.

A functionalized hydrogel coating for wound monitoring was made by immobilizing a fluorescent pH indicator dye, carboxynaphthofluorescein, and a metabolite-sensing enzymatic system, based on GO_x_ and horseradish peroxidase (HRP) on a biocompatible polysaccharide matrix^[64]^, as shown in Figure 5A. The metabolite sensing of glucose is achieved by coupled enzyme reaction, where GO_x_ and HRP react sequentially, producing a substrate for HRP. A change in glucose concentration is converted into a fluorescent signal, allowing glucose monitoring in the wound. This system was able to sense glucose linearly for up to 2.5 mM. In another case of glucose monitoring for wound healing, a multifunctional zwitterionic hydrogel was developed to monitor pH and glucose level, as shown in Figure 5B^[65]^. It consisted of a pH indicator dye (phenol red) and two glucose-sensing enzymes, GO_x_ and HRP, encapsulated in the anti-biofouling and biocompatible zwitterionic poly-carboxy betaine (PCB) hydrogel matrix. Improved activity and stability of the GO_x_ and HRP are observed when present inside the PCB hydrogel. The visible images were captured by a smartphone and transformed into RGB signals to quantify the wound parameters [Figure 5C] and then processed in MATLAB. By using a photographic method to establish functional relation between glucose concentration and RGB intensity, it was found that red and green signals of PCB-PR-E hydrogel increase with increasing glucose concentration while the blue signal is relatively kept constant. Signals were then standardized by dividing the intensity values of each red and green pixel by the referential blue pixel. Therefore, the R/B of each pixel could be obtained and displayed in pseudo color [Figure 5D]. The fitted curve of glucose concentration corresponded to a three-parameter logarithmic equation [Figure 5D]. It was able to monitor glucose levels of 0.1–10 mM.

### Uric acid:

**Figure 6.**
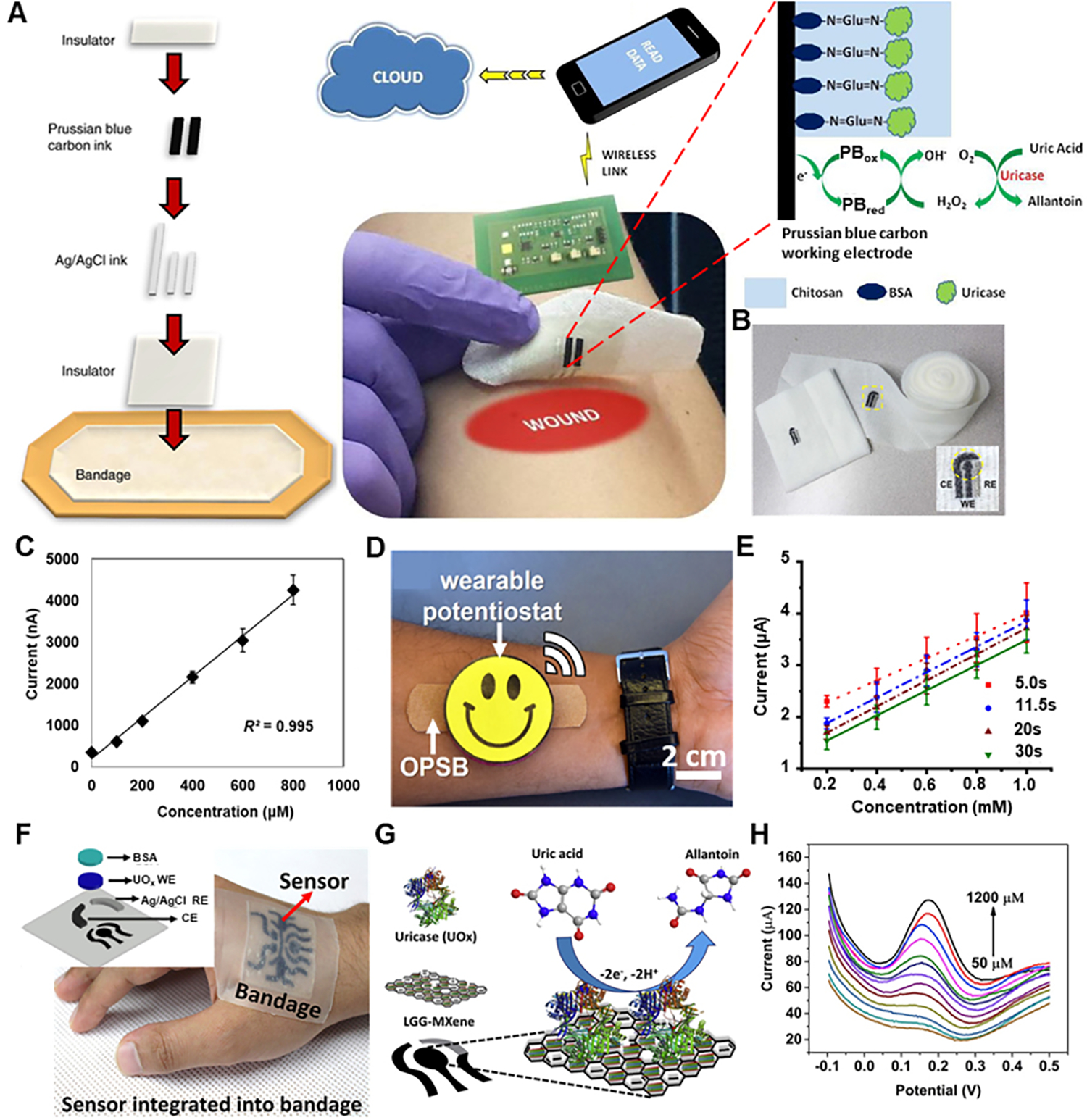
Wearable uric acid-sensing devices for wound healing. (A) Schematic diagram, wireless data transmission, and working mechanism of smart bandage for uric acid sensing. Reproduced with permission^[66]^. Copyright 2015, Elsevier. (B) Embroidered electrochemical sensors on gauze and wound dressing. Inset shows the sensor’s close-up image with the dashed circle representing the sensing region. (C) Calibration plot of generated current vs. uric acid concentration. Reproduced with permission^[67]^. Copyright 2017, Elsevier. (D) Wearable potentiostat interfacing with functional OPSB. (E) Calibration plots of the current as a function of the concentration of uric acid at different reading times. Reproduced with permission^[68]^. Copyright 2018, Elsevier. (F) Photograph of the stretchable and flexible smart bandage embedded on an object’s hand with the inset showing exploded view of the uric acid sensor of the smart bandage. (G) Working mechanism of the uric acid sensor. (H) Uric acid sensor response to variation in uric acid concentration. Reproduced with permission^[61]^. Copyright 2020, Elsevier.

Most uric acid-sensing techniques for wound healing are based on various types of electrochemical measurements. A smart bandage was developed by screen printing an amperometric biosensor directly on a wound dressing for determining uric acid status [Figure 6A]^[66]^. This bandage with biosensor was interfaced with a custom-designed potentiostat that provided wireless data transfer of uric acid status to a computer or smartphone via radio frequency identification (RFID) or near-field communication (NFC). This whole scheme is shown in Figure 6A. Immobilized uricase with a printed Prussian blue transducer facilitates the chronoamperometric detection of uric acid at a low working potential. The enzyme uricase provides highly specific oxidation of uric acid, and the Prussian blue-carbon electrode catalytically reduces the hydrogen peroxide product of uric acid oxidation. This enables sensitive and precise detection of uric acid at a very low negative working potential. The schematic of amperometric detection of uric acid is shown in Figure 6A. To check the performance of the bandage for detecting uric acid, its current response was checked for uric acid at the concentration of 100–800 μM, and its sensitivity was found to be ~−2.4 nA/μM. Similarly, a uric acid biosensor was embroidered on a gauze wound dressing [Figure 6B] for quantitative measurement of the wound marker^[67]^. The threads embroidered as working and counter electrodes were dipped in carbon ink, and the reference electrode thread was dipped in Ag/AgCl ink. The result for a simulated wound fluid spiked with different uric acid concentrations is shown in Figure 6C, thus showing a direct correlation between uric acid concentration and generated electrochemical current. In another attempt at wearable uric acid detection, an omniphobic paper-based smart bandage (OPSB) was fabricated, functionalized with uricase solution, and then interfaced with a reusable wearable potentiostat [Figure 6D]^[68]^. The wearable potentiostat with the OPSB could detect uric acid levels at the wound site and wirelessly transfer data regarding the wound status. Its real-time performance for measuring uric acid concentration was checked, and a linear relationship was found between oxidation current and concentration of uric acid, as shown in Figure 6E. In another case for uric acid sensor, 2D MXene nanosheets were used to functionalize 3D porous laser-guided graphene (LGG) sheets via C-O-Ti covalent crosslinks to obtain LGG-MXene hybrid scaffolds, which were transferred onto polydimethylsiloxane (PDMS) to engineer a high-performance stretchable, flexible multifunctional integrated sensor wound bandage [Figure 6F]. The photograph of the smart bandage is shown in Figure 6F, with the exploded view of the uric acid sensor of the smart bandage shown in the inset of Figure 6F. The uric acid sensor consists of three electrodes: working electrode (LGG-MXene/PDMS), counter electrode (LGG-MXene/PDMS), and reference electrode (Ag/AgCl). The working principle for the uric acid sensor is shown in Figure 6G. The uric acid detection mechanism can be ascribed to the following chemical reactions:

$$\text{UA}+2{\text{H}}_{2}\text{O}+\;\text{Uricase}\;(\text{ox})\to \;\text{Allantoin}\;+{\text{CO}}_{2}+2{\text{H}}^{+}+\text{Uricase}\;(\text{red})$$

$$\text{Uricase}\;\left(\text{red}\right)\;+\;2{\left[Fe(CN)6\right]}^{3-}⇋\text{Uricase}\;(\text{ox})\;+\;2{\left[Fe\left(CN\right)6\right]}^{4-}$$

$$2{\left[Fe\left(CN\right)6\right]}^{4-}⇋2{\left[Fe\left(CN\right)6\right]}^{3-}+\;2e^{-}$$

A linear detection range of 50–1200 μM was observed with a sensitivity of 422.5 μA/mm cm^2^, as shown in Figure 6H.

### Oxygen:

**Figure 7.**
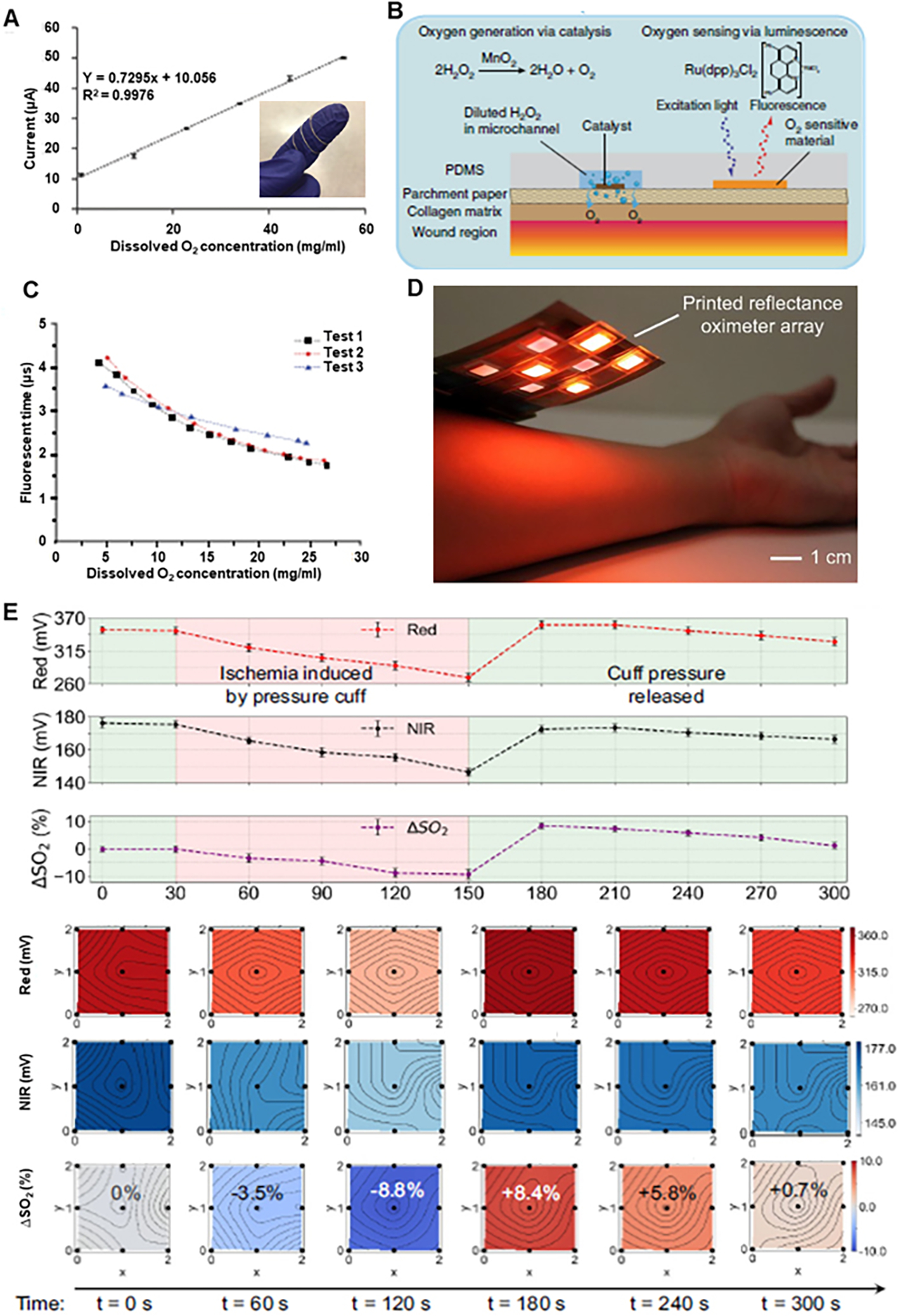
Wearable oxygen sensing devices for wound healing. (A) Calibration plot of wire sensor for dissolved oxygen concentration with inset of the optical image of oxygen sensor looped around finger. Reproduced with permission^[69]^. Copyright 2021, Royal Society of Chemistry. (B) Mechanism for generating and sensing oxygen using flexible smart dressing. (C) Fluorescence lifetime changes with increased dissolved oxygen concentration from 1-layer sensing dye. Reproduced under the terms and conditions of the CC BY 4.0^[70]^. Copyright 2020, The Author(s), published by Nature. (D) Optical image of printed reflectance oximeter array. (E) Saturated Oxygen concentration changes over time, and 2-D contour maps of red, NIR, and saturated oxygen concentration change under normal conditions (*t* = 0 s), under ischemia (*t* = 60, 120 s), and after releasing pressure (*t* = 180, 240, 300 s). Reproduced under the terms and conditions of the CC BY-NC-ND 4.0^[71]^. Copyright 2018, Author(s), published by Proceedings of the National Academy of Sciences USA.

Most wearable electronic devices developed for oxygen sensing for wounds are based on electrochemical sensors, oximetry, fluorescence, etc. A flexible thread-based electrochemical sensor was developed to monitor oxygen^[69]^. This sensor was minimally invasive and had superior flexibility for tissue integration [Figure 7A]. This sensor could detect dissolved oxygen levels within the physiological range in tissues and was insensitive to pH of 5.8–8.0. The reactions taking place for the electrochemical sensors are:

$$\text{Anode}:\;4Ag\;+\;4Cl^{-}{}_{\to }4AgCl\;+\;4e^{-}$$

$$\text{Cathode}:\;4H\;+\;4e^{-}+\;O_{2}\to 2H_{2}O$$

$$\text{Overall}:\;4Ag\;+\;4Cl^{-}+\;4H^{+}+\;O_{2}\to 4AgCl\;+\;2H_{2}O$$

The resulting current passing through the cathode was proportional to the dissolved oxygen concentration. The calibration curve of the wire sensor in the full oxygen range (0%−100% oxygen saturation in phosphate-buffered saline (PBS) solution, or 0–55.47 mg L^−1^ dissolved oxygen) is shown in Figure 7A. In another attempt to sense oxygen, but using fluorescence, a flexible, paper-based biocompatible platform for delivering and sensing oxygen in the wound region was developed with paper being substrate and made using inkjet printing^[70]^. The substrate was patterned with phosphorescent oxygen-sensitive ink based on a ruthenium compound (Ru(dpp)_3_Cl_2_) to enable oxygen sensing. This ink was electronically excited by illumination with light of sufficiently high energy to emit a phosphorescent response. The oxygen molecules quenched the ink and altered the phosphorescence decay rate, from which it is possible to infer the oxygen concentration near the ink. This ink was excitable at wavelengths in the visible spectrum and thus does not require high-energy light (which may affect DNA and other biological compounds in the wound). The sensing mechanism and the structure of the sensor are shown in Figure 7B, and the detailed result for one layer of sensing ink is shown in Figure 7C. Another way for oxygen sensing for wound monitoring has high potential is using oximetry. Keeping this in mind, a large area, 2D reflectance oximeter array based on a flexible and printed organic optoelectronic system [Figure 7D] was developed^[71]^. This oximeter array was composed of four red and four near-infrared (NIR) organic light-emitting diodes and eight organic photodiodes, which sensed reflected light from tissue to determine oxygen saturation. The photodiodes and OLED arrays are fabricated by blade coating and screen printing on separate substrates and then assembled to form an oximeter array. The array implementation allows 2D mapping of oxygenation of an area rather than a single point. The 2D oxygen saturation monitoring results [Figure 7E] for an experiment in which the array was placed on a volunteer’s forearm to monitor changes in oxygen saturation when blood supply was controlled with a pressure cuff show changes in oxygen level when the pressure cuff was tightened and released.

### Moisture:

**Figure 8.**
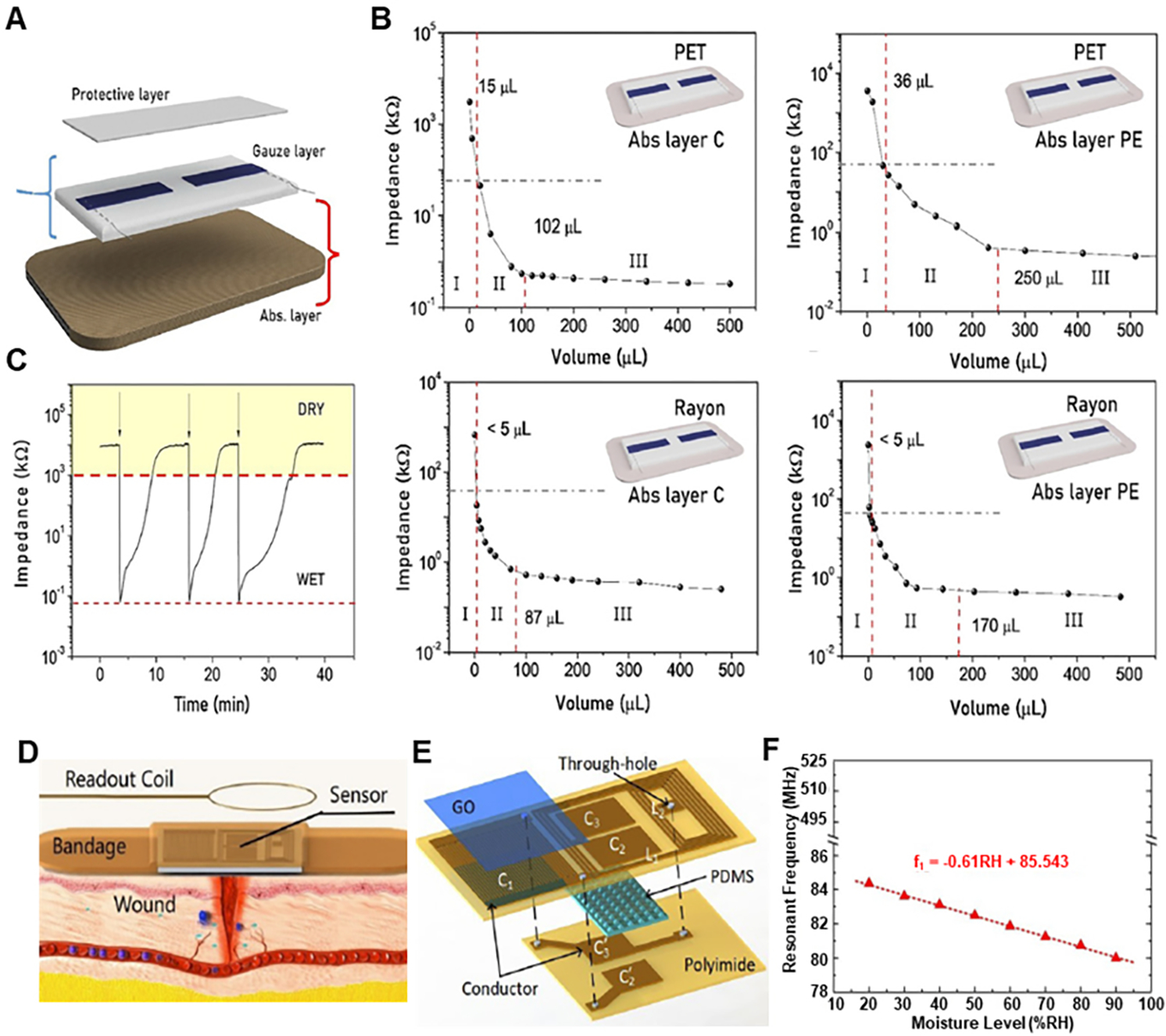
Wearable moisture sensing devices for wound healing. (A) Schematic of bandage sensor showing three different composition layers. (B) Bandage sensor response to the addition of synthetic exudate. (C) Real-time monitoring of the sensor’s impedance when the status switches between wet and dry, showing a reversible behavior. Reproduced under the terms and conditions of the CC BY^[72]^. Copyright 2021, The Author(s), published by Frontiers. (D) Conceptual diagram of wireless moisture sensor integrated with bandage for wound monitoring. (E) Structural diagram of designed LC-type wireless pressure and moisture sensor. (F) Measured resonant frequencies when RH changes from 20% to 90%. Reproduced with permission^[73]^. Copyright 2018, IEEE.

Most research for moisture sensing for wound monitoring purposes has been based on impedance measurement and capacitive measurement. A textile sensor based on a conductive polymer, PEDOT:PSS [Figure 8A], was developed that monitors wound’s moisture level by monitoring impedance variations that span over several orders of magnitude between dry and wet states^[72]^. The sensor was fabricated by depositing two straight non-consecutive PEDOT:PSS strips on to medical gauze dressing, and the electrical contacts were formed by two stainless steel conductive threads sewn onto the PEDOT:PSS coating and then heat sealing the whole setup in a stacked structure of three textile layers: protective, active gauze and absorbing layer. Two sensing textile-based substrates, “Gauze rayon” which is made of 70% rayon and 30% polyethylene terephthalate (PET), and “Gauze PET” which is 100% PET, were considered, and two different absorbing layers were considered. The impedance value for different active substrates with different absorbance layers to different simulated wound exudate values are shown in Figure 8B. This sensor exhibited good reversible behavior for moisture sensing, as shown in Figure 8C. Another way of sensing moisture for wound monitoring which was developed used interdigital capacitors with graphene oxide as moisture sensing material^[73]^. A schematic diagram showing the sensor embedded in the commercial bandage strip acting as a cover package has been shown in Figure 8D. This whole sensor package was fabricated over a flexible polyimide substrate, and it consisted of an interdigital capacitor (C_1_ = X) functionalized by graphene oxide, two symmetrical parallel capacitors (C_2_ = C_3_ = Y) and two equal inductors (L_1_ = L_2_ = L). The structural diagram is shown in Figure 8E. C_1_ is the moisture sensing capacitor. Testing results showed that the moisture sensing frequency of the sensor gives a sensitivity of 61 kHz/%RH in the humidity monitoring range of 20%−90% RH. Detailed results are shown in Figure 8F.

### Proteins:

**Figure 9.**
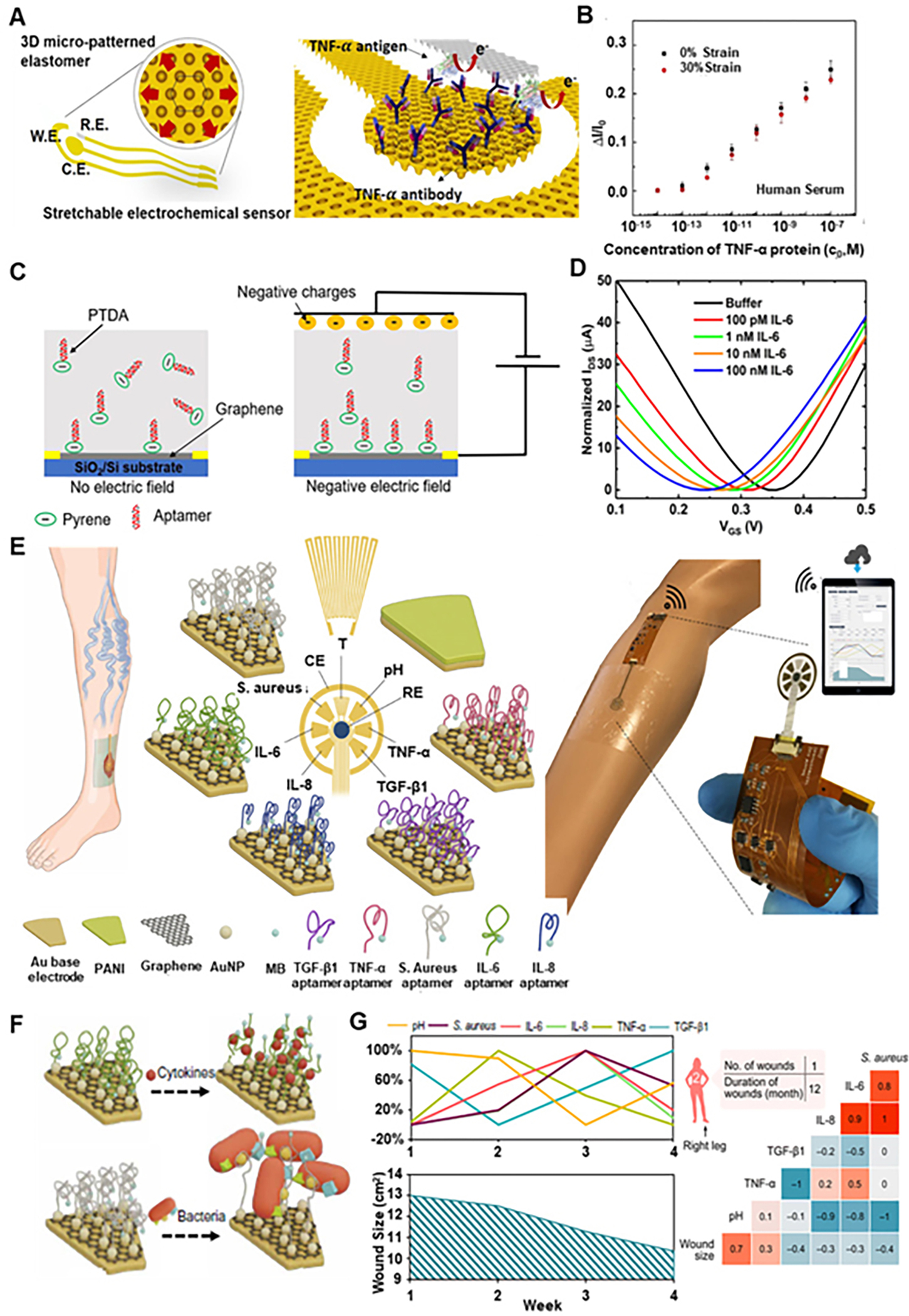
Wearable sensors for proteins. (A) A scheme of a stretchable electrochemical immunosensor fabricated on a three-dimensional (3D) micro-patterned elastomeric substrate and probe proteins (TNF-α Ab) immobilized on the working electrode. (B) Calibration curves of the TNF-α immunosensor (*n* = 3) measured in the undiluted human serum. Reproduced with permission^[74]^. Copyright 2018, Elsevier. (C) Schematic illustration of aptamer-based graphene field effect transistor (GFET) for sensing IL-6 with the effect of the applied external electric field. (D) Transfer curves of GFET sensor when exposed to varying concentrations of IL-6. Reproduced under the terms and conditions of the CC BY^[76]^. Copyright 2021, Author(s), published by MDPI. (E) Illustration of a biomarker analytical dressing applied onto an open wound of a patient and schematic of the immunosensor for detection of TNF-α, IL-6, IL-8, TGF-β1, *S. aureus*, pH, and temperature. PANI: Polyaniline; MB: methylene blue; RE: reference electrode; CE: counter electrode. (F) Schematic of the sensing mechanism of the aptasensors for cytokine and bacteria detection, respectively. (G) Weekly assessment of pH, *S. aureus*, IL-6, IL-8, TNF- α, and TGF- β1 by the immunosensor for each patient and correlation matrices of parameters assessed by the immunosensor (pH, *S. aureus*, IL-6, IL-8, TNF- α, and TGF- β1) and the wound size over a 5-week period. Reproduced with permission^[77]^. Copyright 2021, American Association for the Advancement of Science.

Currently, immunoaffinity (aptamers, antibodies, etc.) are utilized for proteins and immobilized over the electrochemical biosensor as a probe. A high-performance, stretchable and disposable electrochemical biosensor with TNF antibody (abs) as sensor receptor was developed^[74]^, for sensing TNF-α protein [Figure 9A]. This electrochemical sensor was fabricated by depositing Au working and counter electrodes and a stencil printed Ag/AgCl reference electrode on a three-dimensionally (3D) micro-patterned PDMS substrate with continuous curvilinear bumps and valleys. Differential pulse voltammetry was used for electrochemical signal acquisition and this sensor showed stable performance under 1000 cycles of stretching and 30% elongation without losing its electrochemical characteristics. It detected TNF-α in PBS solution down to the 100 fM level with a dynamic range of 10^6^ under no stretching and 30% stretching. This device worked well and showed a linear relationship under no stretching and applied 30% stretching for the TNF-α concentrations from 1 pM to 100 nM in human serum as artificial wound exudate [Figure 9B]. In antigens, engineering them to contain affinity and signal-transducing moieties into the same molecule is not possible; however, this can be done using aptamers. Considering the aptamer immobilization approach, Aptamers are DNA or RNA oligonucleotides designed to bind to various biomolecules with high specificity and sensitivity. Using this aptamer immobilization approach, an electrochemical aptasensor was developed with methylene blue(MB) as a redox label for detecting TNF-α^[75]^. It had a detection limit of 10 ng/ml and could sense up to 100 ng/ml in the linear range. In a similar attempt but for detecting IL-6, a graphene field-effect transistor (GFET) based biosensor was developed^[76]^ using pyrene-tagged DNA aptamer s (PTDA) [ Figure 9C] and with an additional application of an external electric field during the functionalization step, the efficiency of the aptamer immobilization was increased, hence improved coverage and density. The results showed that the sensor is selective to the targeted protein IL-6 and could detect IL-6 concentration from 100 pM to 100 nM in PBS solution [Figure 9D]. Now improving upon this, aptamer functionalized graphene-gold electrodes were integrated into a patch with microfluidic exudate collector [Figure 9E]^[77]^. Few cytokines (IL-6, IL-8, TNF-α, TGF-β1, etc.) were selected for wound monitoring. The sensing mechanism for the cytokines is as follows: in the absence of a target, MB was in proximity to the AuNPs-GP-modified electrodes, allowing electron transfer. A faradaic current could be detected electrochemically. Upon target binding, the hairpin structure of the aptamer went through a conformational change during which MB moved away from the electrodes, causing a decreased redox current. This mechanism is shown in Figure 9F. As a proof of principle, this system was applied to assess wound exudates collected from patients with non-healing ulcers once a week for five consecutive weeks^[77]^. The sensor-derived data analysis for one of the patients is shown in Figure 9G, from which it is clear that the readings of the biomarker varied throughout the study. From all these results, it can be concluded that these multiple biomarker profiles can help finalize the clinical decisions on modality and duration of treatment.

## WEARABLE ELECTRONICS FOR ACCELERATED WOUND HEALING

### Wearable electrical stimulators

**Figure 10.**
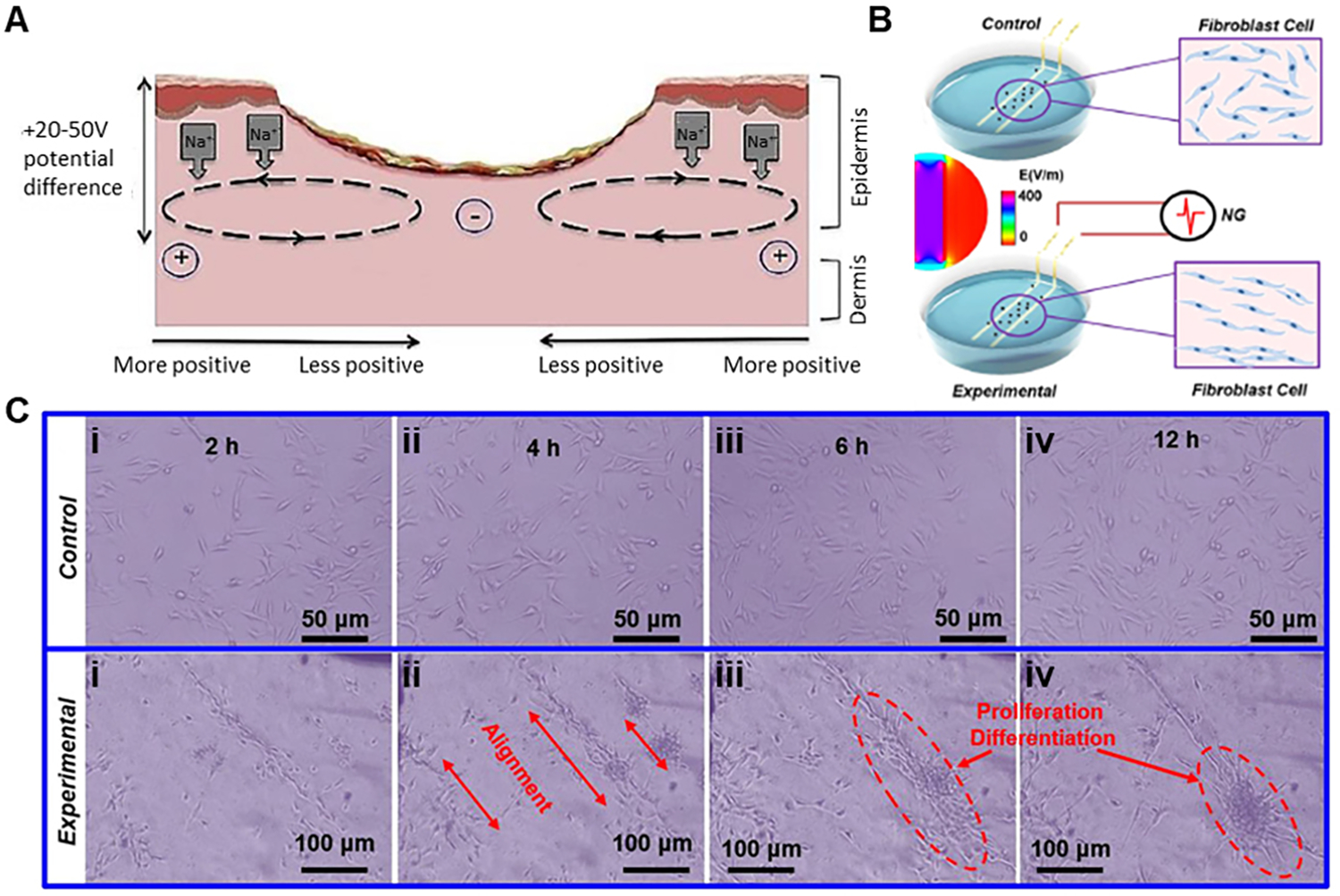
Mechanism for wound healing by electrical stimulation. (A) Current flow through the wound. Reproduced under the terms and conditions of the CC BY^[83]^. Copyright 2021, Author(s), published by MDPI. (B) Schematic image of cells cultured in a dish with Au electrodes connected and disconnected to NG generated pulse voltage. Middle inset is the simulated electric field distribution in the culture dish when the electrodes are connected to an NG with ± 2 V out voltage. (C) Cultured cell morphology at different time points without (control) and with (experimental) electrical stimulation. Reproduced with permission under the terms of the Standard ACS Author Choice/Editors’ Choice usage agreement^[85]^. Copyright 2018, American Chemical Society.

The wound healing process is influenced by the skin’s endogenous electric potential^[78]^. A natural electric potential of 10–60 mV exists in undamaged skin between the epidermal and sub-epidermal layers^[79]^. This is due to the transport of ions through ion channels and pumps^[80]^. The disruption in the epithelium creates a short circuit to the TEP, causing positive potential flow towards the wound, as shown in Figure 10A^[81–83]^. Studies have shown the voltage difference between wound site and undamaged skin to be around 100–150 mV/mm^[80–82]^. These endogenous electric fields are essential for wound healing, and the resulting endogenous currents act as a cue for cellular migration, thus helping wound healing. Wound edge keratinocytes migrate to the cathodal pole, which directs them to the center of the wound^[81,84]^. Fibroblast growth was studied under electrical stimulation of ± 2 V output voltage^[85]^ [Figure 10B]. The fibroblast cells aligned along the direction of the electric field and later proliferated and differentiated [Figure 10C]^[86]^. Wearable electrical stimulators based on triboelectric nanogenerators have exploited the aforementioned phenomenon.

**Figure 11.**
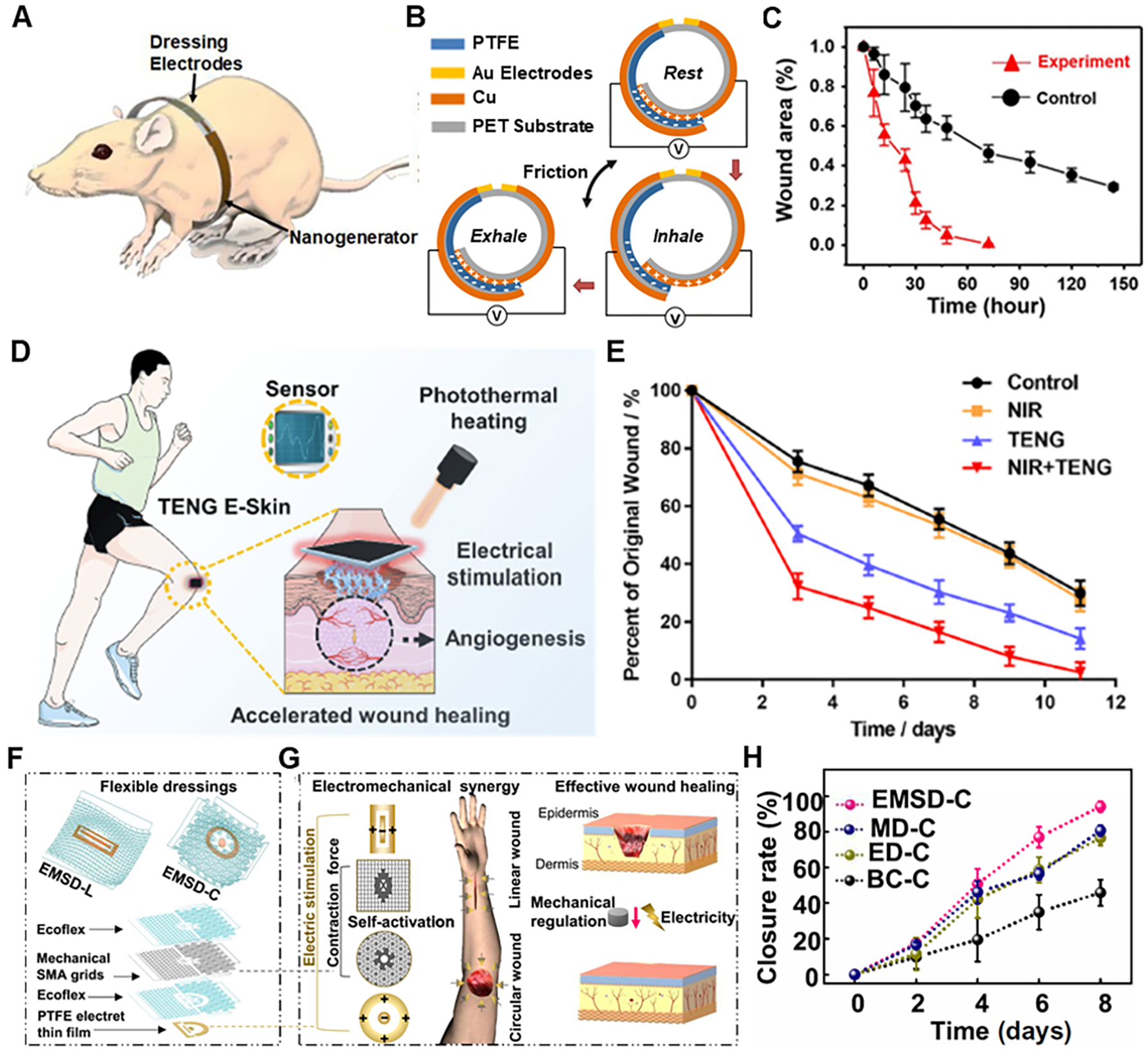
Wearable electrical stimulators for wound healing. (A) Biomechanical energy harvesting of NG by rat breathing using bandage wrapped around rat. (B) Schematic and mechanism of energy harvesting by bandage during breathing. (C) Wound area as a function of time with (red curve) and without (black curve) electric field stimulation. Reproduced with permission under the terms of the Standard ACS Author Choice/Editors’ Choice usage agreement^[84]^. Copyright 2018, American Chemical Society. (D) Functions of the wearable TENG E-skin patches in the wound: NIR photothermal heating, electrical stimulation, and sensing. (E) Quantification of wound closure rates. Reproduced with permission^[90]^. Copyright 2021, Elsevier. (F) Schematics of the overall EMSD-L and EMSD-C (top). Exploded illustration of the device components, essential materials, and multilayer structures (bottom). (G) Working principle of wound treatment by programmable and skin temperature–activated EMSDs. (H) Wound closure rate over time of the wound area from ESMD-C, SMA-based mechanical dressing (MD-C), Endogenous electric field-based electrical dressing (ED-C), and blank control group (BC-C), respectively. Reproduced with permission^[91]^. Copyright 2022, American Association for the Advancement of Science.

A self-powered electrical wound bandage to accelerate wound healing was developed [Figure 11A]^[84]^. With this bandage, the kinetic energy generated by a rat breathing was converted into discrete alternating current voltage signal through the patch, and then the electric field was applied to the wound. This bandage consisted of two parts: the biomechanical energy conversion part [triboelectric nanogenerator (TENG) part], which consisted of overlapping of Cu/PTFE (electronegative material) layer by another Cu (electropositive material) layer on different sides of PET substrate and the dressing electrodes [Figure 11B]. The PTFE layer could slide in between the two Cu layers and, due to the breathing of the rat, caused a change in the overlapping area between the electropositive and electronegative layers, driving charge flow toward or away from the dressing electrodes and thus, induced an alternating electric field between the electrodes [Figure 11B]. The generated electric field reached different voltage levels corresponding to different breathing patterns of the rats, ranging from 0.2 to 2.2 V. To prove the accelerated wound healing by the electrical bandage, rat studies were conducted for full-thickness rectangular skin wounds, which rapidly healed within three days compared to 12 days of rodents’ usual contraction-based healing process. The described wound healing results compared to wound healing without electrical stimulation are shown in Figure 11C. However, there are some limitations to this system, for example, mechanical discontinuity due to irregular contact between the plate electrodes can cause improper contact between the TENG surface and wound surface, hence weakening the endogenous electric field. A possible solution for this problem can be a wearable ionic TENG healing device consisting of a fully stretchable wearable ionic TENG patch, a wire, and a multifunctional biocompatible ionic patch encapsulated with the elastomeric film serving as both electrode and wound dressing. It was characterized by a flexible gel-based platform for better contact between wound surface and TENG patch, developed to harvest biomechanical energy and deliver electrical stimulation to injured tissue^[87]^. Some other similar examples include a flexible, stretchable sandwich structured triboelectric patch composed of double-layer polydimethylsiloxane (PDMS) membrane with a PAAm-LiCl hydrogel embedded^[88]^ and genetically engineered recombinant spider silk proteins based TENG patch^[89]^. To improve upon the TENG concept of wound healing as described above, a single-electrode TENG-based e-skin patch for promoting wound healing by synergistically utilizing electrical stimulation and photothermal heating capability, with conductive and photothermal polypyrrole/Pluronic F127 hydrogels as electrolytes^[90]^ was developed, and the whole concept is shown in Figure 11D. This patch showed promising results by combining photothermal heating and real-time electrical stimulation, thus promoting angiogenesis, collagen deposition, and re-epithelization to accelerate tissue regeneration and wound closure in a shorter period of ~9 days, as shown in Figure 11E. Besides wearable electrical stimulators based on TENG, there are other approaches to electrical stimulators for wound healing.

One such way of electrical stimulation for wound healing was using stretchable bioelectric plaster with built-in enzymatic biofuel cell (EFBC)^[91–94]^ that conforms to skin and generates ionic current along the skin’s surface by enzymatic electrochemical reactions for more than 12 h. To improve upon these attempts for wound healing using electrical stimulation, a programmable and skin temperature-activated electromechanical synergistic wound dressing (ESMD) [Figure 11F] composed of a shape memory alloy-based mechanical metamaterial for wound contraction and an antibacterial electret thin film for electric field generation was developed, which showed promising results for linear and circular wounds, respectively with over 50% improvement in wound closure as compared to the blank control group^[95]^. The working principle of ESMD is shown in Figure 11G. The ESMD was applied in a stretched state over the wound surface, and as the temperature of the wound changed, it triggered the stretched ESMD to restore to the initial state and ensure contraction force on the wound. Also, the electret thin-film ensured electric field generation for electrical stimulation of the wound for faster healing. The described wound healing results for the ESMD compared to only mechanical contraction, only electric field, and blank control group for the wound healing are shown in Figure 11H.

### Wearable light stimulators

There have been two main light stimulation therapies for wound healing. The first is photobiomodulation (PBM). It has been defined as “a form of light treatment that utilizes nonionizing forms of light sources, including lasers, light-emitting diodes (LEDs), and broadband light, in the visible and infrared spectrum, involving a nonthermal process with endogenous chromophores eliciting photophysical (i.e., linear and nonlinear) and photochemical events at various biological scales. This treatment results in beneficial therapeutic outcomes including, but not limited to, the alleviation of pain or inflammation, immunomodulation, and promotion of wound healing and tissue regeneration”^[86,96,97]^. The second one is photodynamic therapy (PDT). This therapy involves the delivery of light-emitting at a specific wavelength to a photosensitizer, which is administered either systematically or topically to the surface that needs treatment. Upon excitation of photosensitizer, a material with high absorption and high intersystem crossing rate to a triplet state, triplet excitons interact with molecular oxygen present in the tissue, lifting oxygen from its triplet ground state into the singlet state. These singlet oxygen species and created free radicals are cytotoxic and attack nearby cells and bacteria with spatiotemporal selectivity^[98]^. Considering both these light stimulation therapies, wearable light stimulators have been developed for wound healing.

**Figure 12.**
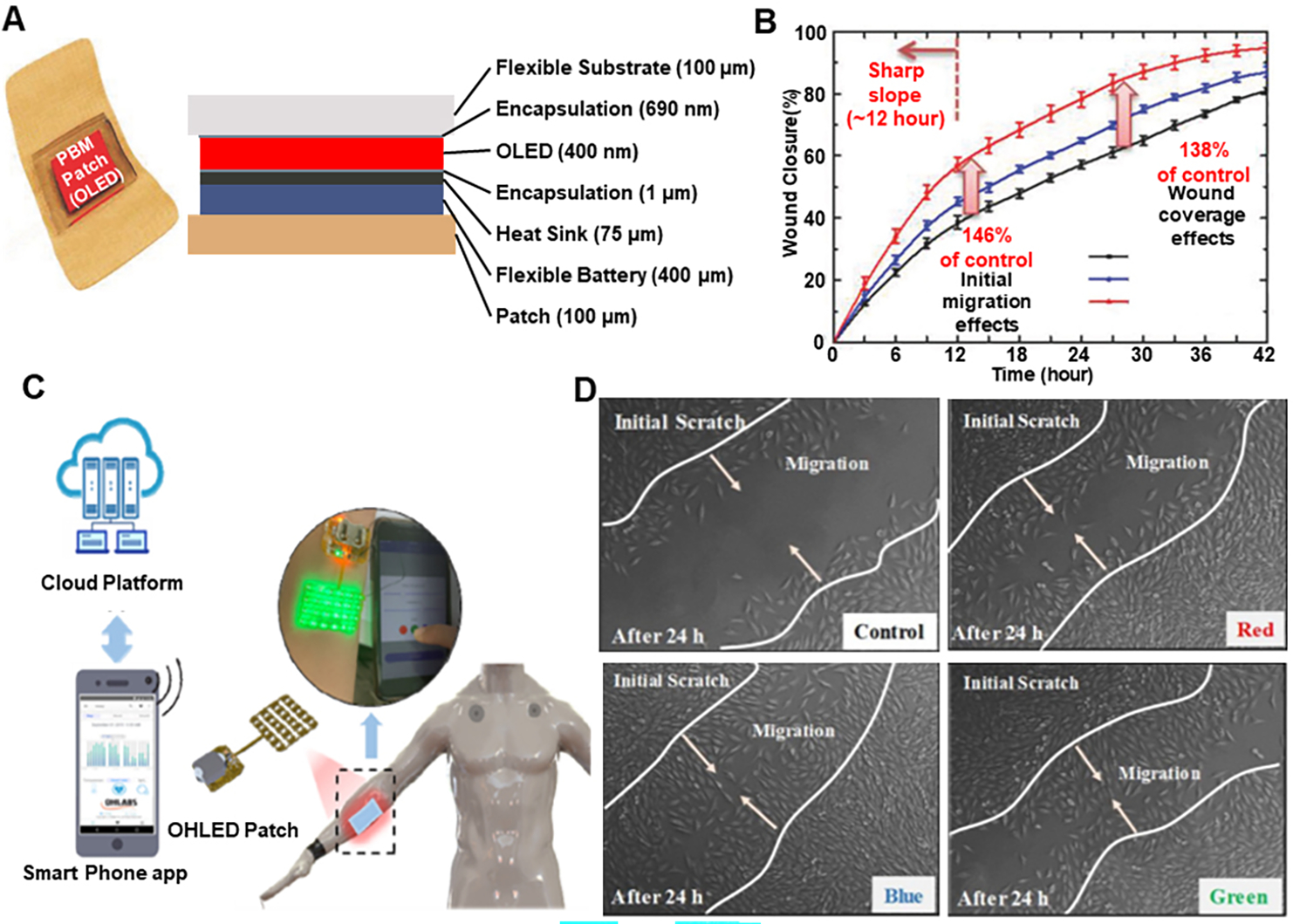
Wearable devices for light stimulation (photobiomodulation) for wound healing. (A) Structure of the wearable PBM patch (B) Wound healing effect depending on the wavelength and energy of the OLEDs. Reproduced with permission^[99]^. Copyright 2018, WILEY-VCH Verlag GmbH & Co. KGaA, Weinheim (C) Design of wearable LED patch. (D) Wound healing effect depends on the wavelength and energy of the LED. Reproduced under the terms and conditions of the CC BY^[100]^. Copyright 2022, Author(s), published by MDPI.

Considering photobiomodulation, a wearable PBM patch [Figure 12A] was developed, which used a flexible red-wavelength organic light-emitting diode (OLED) source, which could be attached to the human body^[99]^. This patch consisted of flexible OLED modules and a battery module with a patch to allow skin attachment. Also, to check the wound healing effects of the OLED PBM system, an artificial wound was made, the wound was irradiated under the various wavelength of OLED, and wound closure percentage was monitored over time. The results for 10 min of irradiation for 630 nm and 650 nm are shown in Figure 12B, where it is proved that this method leads to wound healing acceleration. In another attempt to make flexible PBM device, a flexible and wireless LED therapy patch [Figure 12C] was developed for skin wound healing with IoT-connected healthcare applications^[100]^. It was proven that this patch had wound-healing effects when I929 fibroblast cells were treated with various color lights, as shown in Figure 12D, where the lights accelerated cell migration. Considering augmentation of effects of photobiomodulation for wound healing, an adhesive hyaluronic acid-based gelatin nanofibrous membranes integrated with multiple light-emitting diode (LED) arrays were developed as a skin-attachable patch^[101]^. The nanofibrous wound dressing mimicked the three-dimensional structure of the ECM, and its adhesiveness allowed tight coupling between the wound sites and the flexible LED patch. Experimental results demonstrated that this medical device accelerated the initial wound healing process through the synergetic effects of the wound dressing and LED irradiation.

**Figure 13.**
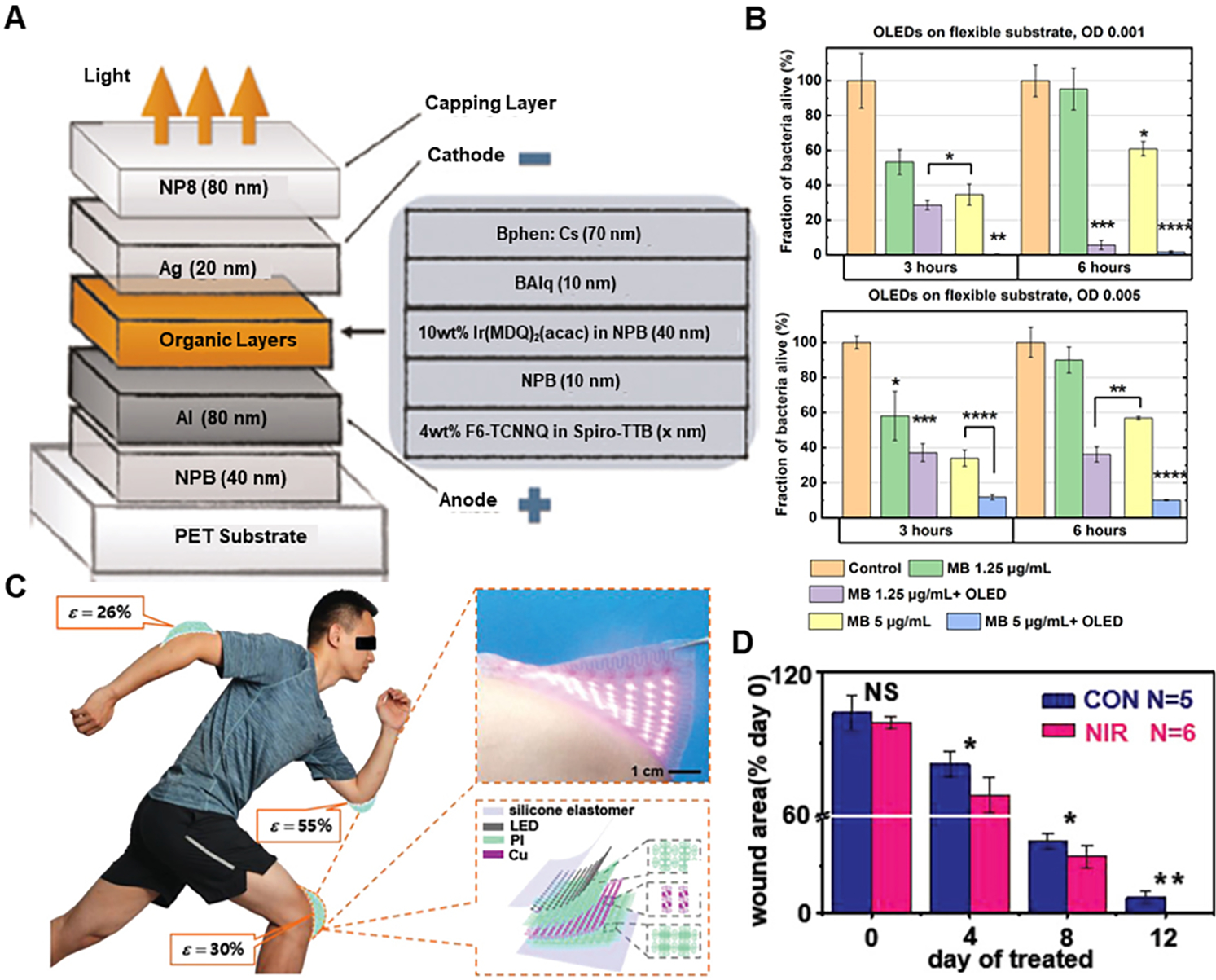
Wearable devices for light stimulation (Photodynamic Therapy) for wound healing. (A) Device structure of flexible p-i-n OLED. (B) Biological results of OLED-PDT for killing *S. aureus*. Reproduced under the terms and conditions of the CC BY 4.0^[102]^. Copyright 2019, The Author(s), published by Springer Nature. (C) The ISASE for wound healing. (D) Wound closure analysis in rat by the ISASE. Reproduced with permission^[103]^. Copyright 2019, WILEY-VCH Verlag GmbH & Co. KGaA, Weinheim.

Considering PDT, there has been significant research for developing wearable devices to use this therapy. Flexible top-emitting OLEDs have been developed^[102]^ with the ability to tune the emission peak from 699 nm to 737 nm to match the photosensitizer MB, with high irradiance and low driving voltage. All these features make it ideal for ambulatory PDT. The schematic of the device is shown in Figure 13A. To prove its wound-healing effects, it was used to kill Staphylococcus aureus bacteria in a relatively short time, accompanied by MB. The fraction of bacteria alive for different bacterial concentrations on flexible substrates is shown in Figure 13B, which shows more than 90% bacterial death for the flexible substrate. In another attempt, a novel infrared skin-like active stretchable electronics (ISASE) [Figure 13C] was introduced with the organic-inorganic composite design for promotion of cutaneous wound healing, which can be conformally mounted anywhere on the human body due to its excellent flexibility and stretchability^[103]^. By combining stretchable electric circuits with the NIR LED at 850 nm wavelength, ISASE promoted active acceleration of wound healing in a portable and wearable way. With the organic-inorganic composite design of the structure, the device had very high electronic performance, low heat generation, low mechanical stiffness, and high elastic deformability under large strain. The experiments for cure functions to treat the cells under optimized NIR radiation dose showed that the ISASE effectively promotes the migration and proliferation of cells by irradiation of human skin fibroblasts (HSFs) and rat dermal fibroblasts (RDFs). As shown in Figure 13D, Wound closure analysis shows the wound healing effects on an artificial wound created on a rat, proving this PDT device’s accelerated wound healing effects.

### Wearable intelligent drug delivery systems

Wound patches/dressings combining biosensors with biomarker-responsive drug release systems have become a novel way of accelerating wound healing. These patches can monitor the wound status in real-time and perform drug release simultaneously.

**Figure 14.**
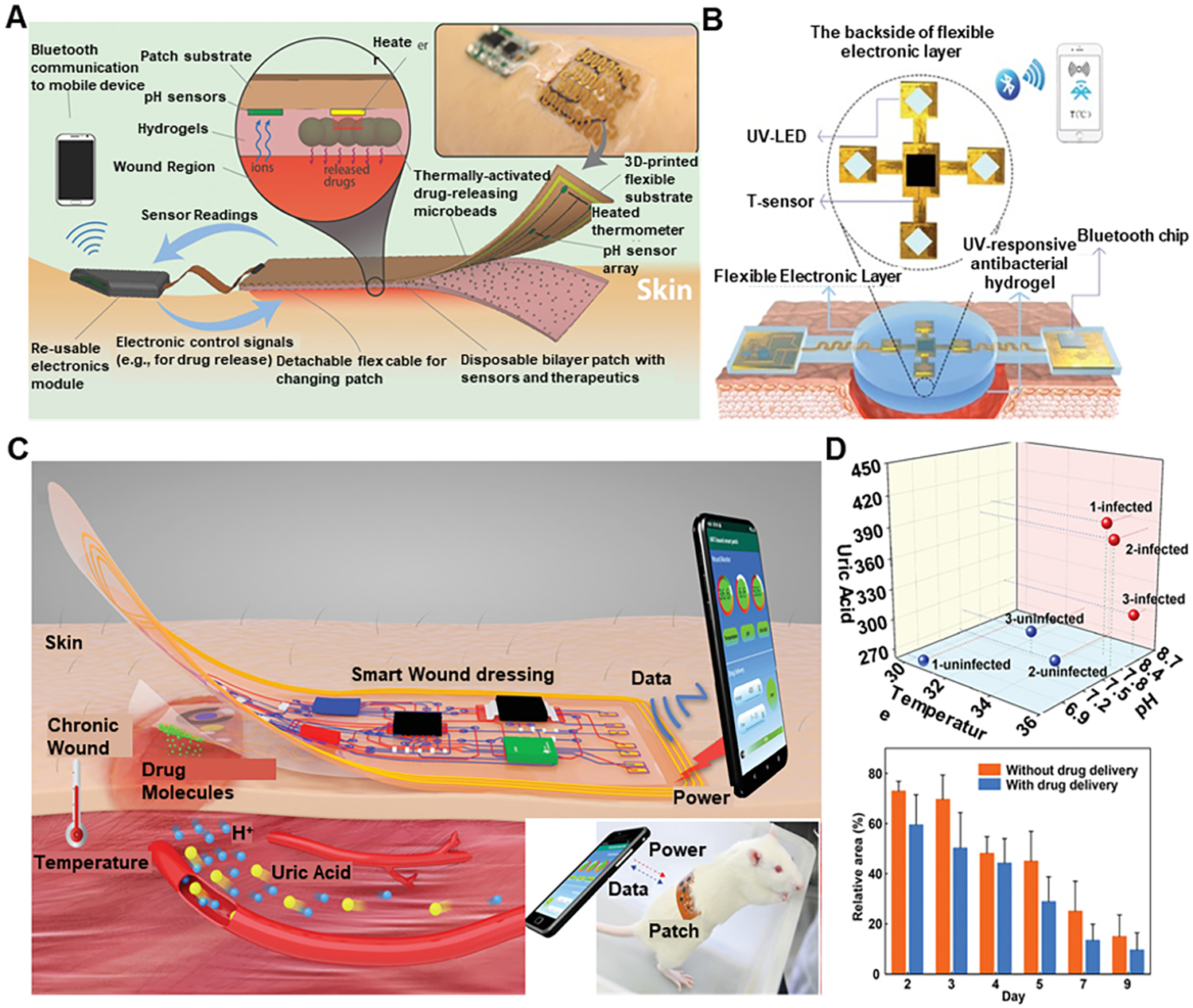
Wearable devices for smart drug delivery. (A) Schematic and conceptual view of the automated smart bandage. Reproduced with permission^[104]^. Copyright 2018, WILEY-VCH Verlag GmbH & Co. KGaA, Weinheim. (B) Schematics of the structures of the smart, flexible electronics-integrated wound dressing. Reproduced under the terms and conditions of the CC BY^[105]^. Copyright 2020, Author(s), published by WILEY-VCH Verlag GmbH & Co. KGaA, Weinheim. (C) The battery-free and wireless wound dressing for wound infection monitoring and electrically controlled on-demand drug delivery with the inset showing the dressing (patch) over a rat. (D) In situ animal studies on wound monitoring and infection treatment. Reproduced with permission^[106]^. Copyright 2021, Wiley-VCH GmbH.

An intelligent dressing [Figure 14A] was engineered that utilized electrochemical sensor for measuring pH and temperature, and with an onboard controller, pH data were processed to detect potential pH values indicating infection^[104]^. This controller can automatically trigger the integrated, flexible heater to heat the hydrogel-carrying drug to release the antibiotics. Another intelligent patch that uses temperature as a biomarker for wound healing integrated with a drug delivery system [Figure 14B] was developed^[105]^. This system consisted of a double-layer structure, with the first layer consisting of PDMS encapsulated flexible temperature sensor and ultraviolet (UV) light-emitting diodes and the other layer consisting of a UV-responsive antibacterial hydrogel. When the temperature was above the present safe limit, the smartphone can control ultraviolet light exposure via Bluetooth to release the drugs. An animal experiment was conducted to prove its working to demonstrate that this system can monitor wound status in real-time, detect bacterial infection, and provide needed treatment. Another example of such closed-loop monitoring and treatment system is shown in Figure 14C^[106]^. This system consisted of a fully integrated, battery-free, flexible and wireless smart wound dressing for wound infection detection and on-demand drug delivery and is flexible. This dressing is designed to be a double-layer patch, including a disposable sensing layer and a reusable circuit layer. The smart wound dressing integrated with the near field communication module could realize wireless power harvest and data transmission, on-site signal processing, and drug delivery control through the miniaturized circuit and smartphone. The wound’s temperature, pH, and uric acid were detected simultaneously by the developed sensors to assess wound conditions. Meanwhile, the drug delivery electrode in the dressing was used to provide on-demand infection treatment by delivering the electrically controlled antibiotic. Through *in situ* animal studies [Figure 14D], it is shown that the dressing can effectively accelerate wound healing, thus validating its effectiveness in wound treatment.

## CONCLUSION

Conventional wound assessments are based on regular visual evaluations of the wound bed and the surrounding skin. A combination of real-time wound monitoring, intelligent clinical decision making, and controlled intelligent treatment of the wound is expected to benefit wound management and speed up healing. For real-time monitoring of wound status, combining wearable sensors with wireless data transmission techniques to detect a wide variety of physical and chemical biomarkers (pH, temperature, glucose, uric acid, etc.) is a promising method. Also, development in material technologies has enabled wearable sensors to detect the biomarkers for a longer time without a decline in performance.

Despite all these developments in wound care management using wearable electronics, many obstacles still need to be addressed. Some challenges are: (1) When an early infection occurs, physiological changes in the wound site are more subtle and susceptible to external noise like human movement. As such, it is required that the performance parameters (sensitivity, accuracy, response time, etc.) of the next generation of wearable electronic devices be improved; (2) There has been limited research on wearable sensors for detecting proteins in the wound bed. Since infection is a key challenge and can even lead to life-threatening conditions, wearable sensing devices that can directly measure bacterial load would be extremely useful; (3) Skin and skin wounds are elastic tissues with complex surface morphologies. Hair follicles and hair protrusions cover the skin surface, and the wound fluids may be secreted on this surface. Also, the wound area has complex morphological characteristics and a complicated humidity environment. Due to these factors, steady adhesion of wearable devices/patches for a prolonged period is difficult. So, the wearable device/patch needs to steadily adhere to the skin for a long time on both dry skin and wet wound areas; (4) Most wearable electronics for wound care have mainly been tested *in vitro*. Very few human trials have been performed using these wearable electronic devices.

Overall, these intelligent devices/patches based on wearable sensors and treatment platforms have shown great potential in wound management. The ultimate goal for these wearable intelligent devices/patches is to monitor the wound status in real time and ensure a better recovery, decreasing patients’ visits to hospitals and mitigating their financial burden. Possible solutions discussed in this review can create new opportunities for further developing wearable intelligent devices for more accurate and efficient wound care management.

## Data Availability

Not applicable.
